# SIV Nef Proteins Recruit the AP-2 Complex to Antagonize Tetherin and Facilitate Virion Release

**DOI:** 10.1371/journal.ppat.1002039

**Published:** 2011-05-19

**Authors:** Fengwen Zhang, Wilmina N. Landford, Melinda Ng, Matthew W. McNatt, Paul D. Bieniasz, Theodora Hatziioannou

**Affiliations:** 1 Aaron Diamond AIDS Research Center, The Rockefeller University, New York, New York, United States of America; 2 Laboratory of Retrovirology, The Rockefeller University, New York, New York, United States of America; 3 Howard Hughes Medical Institute, The Rockefeller University, New York, New York, United States of America; Harvard University, United States of America

## Abstract

Lentiviral Nef proteins have multiple functions and are important for viral pathogenesis. Recently, Nef proteins from many simian immunodefiency viruses were shown to antagonize a cellular antiviral protein, named Tetherin, that blocks release of viral particles from the cell surface. However, the mechanism by which Nef antagonizes Tetherin is unknown. Here, using related Nef proteins that differ in their ability to antagonize Tetherin, we identify three amino-acids in the C-terminal domain of Nef that are critical specifically for its ability to antagonize Tetherin. Additionally, divergent Nef proteins bind to the AP-2 clathrin adaptor complex, and we show that residues important for this interaction are required for Tetherin antagonism, downregulation of Tetherin from the cell surface and removal of Tetherin from sites of particle assembly. Accordingly, depletion of AP-2 using RNA interference impairs the ability of Nef to antagonize Tetherin, demonstrating that AP-2 recruitment is required for Nef proteins to counteract this antiviral protein.

## Author Summary

Primate lentiviruses express several small proteins which antagonize cellular proteins that inhibit virus replication. One such viral protein, Nef, has recently been shown to antagonize the cellular protein Tetherin that prevents newly formed viral particles from leaving the surface of infected cells. In this study we reveal the mechanism by which Nef overcomes inhibition by Tetherin. We show that three amino acids in the Nef C-terminal flexible loop are important for Tetherin antagonism. We also show that the interaction between Nef and AP-2 adaptor complexes is important for Tetherin downregulation from the cell surface, removal from sites of particle assembly and antagonism. Thus, our study demonstrates that AP-2 is important for the ability of Nef to antagonize Tetherin.

## Introduction

Human and simian immunodeficiency viruses encode several small, so called ‘accessory’, proteins that do not appear to be required for viral replication in most *in vitro* replication systems. Nevertheless, it has become apparent that several of these accessory proteins play important roles in antagonizing host proteins, known as restriction factors, that inhibit viral replication. Specifically, Vif antagonizes members of the APOBEC3 family of cytidine deaminases whereas Vpu and Nef antagonize Tetherin (reviewed in [1]). There is also emerging evidence suggesting that Vpx might also antagonize yet unidentified host restriction factors [2]–[4].

Tetherin (BST-2/CD317/HM1.24) is a cell surface membrane protein with an unusual topology, consisting of a short N-terminal cytoplasmic tail (CT), a transmembrane domain (TM), an extracellular coiled-coil and a glycophosphatidyl inositol anchor at the C-terminus [5]–[8]. This topology, rather than primary sequence appears key for Tetherin's ability to retain nascent mature viral particles at the cell membrane [9]. Indeed, an artificial Tetherin assembled from domains of heterologous proteins with no sequence homology to natural Tetherins is active [9]. Tetherin appears to work by inserting either of its membrane anchors into the lipid envelope of nascent virions. In so doing, it physically bridges the nascent virion and cellular plasma membranes thereby preventing virions from disseminating to infect other target cells [10], [11]. Thus, the spectrum of activity of Tetherin proteins against enveloped viruses is broad and includes retroviruses, filoviruses, arenaviruses, rhabdoviruses and herpes viruses [12]–[17].

Perhaps because Tetherin targets the lipid envelope, an almost invariant component of the virion, to block particle release, divergent viruses evolved various strategies and proteins to counteract Tetherin. HIV-1 uses Vpu, a type-I transmembrane protein [10], [11], Ebola uses its envelope protein [13] and Kaposi's sarcoma-associated herpesvirus uses the viral RING-CH E3 ubiquitin ligase K5 [14], [15]. Interestingly, even among relatively closely related primate lentiviruses, three different viral proteins (Vpu, Nef and Env) have assumed the function of Tetherin antagonism [10], [11], [18]–[21]. Vpu is encoded by a subset of primate lentiviruses including HIV-1, its direct chimpanzee-derived ancestor (SIVcpz) and the SIVgsn/mus/mon lineage whose 3′ portion of the genome, including Vpu, shares a common origin with SIVcpz/HIV-1 [22]. Vpu proteins from HIV-1 and SIVgsn/mus/mon antagonize Tetherin proteins from their respective hosts [20], [23], [24]. Even though both its direct descendent (HIV-1) and its ancestors (the SIVgsn/mus/mon lineage) use Vpu to antagonise Tetherin, SIVcpz instead employs Nef for this function. In fact with the exception of HIV-2 [19], that uses the envelope glycoprotein, all other primate that lack Vpu and have been tested encode Nef proteins that can counteract Tetherin [18], [20], [21], [24].

The diverse nature of Tetherin antagonists that have arisen in primate lentiviruses is a consequence of the diversity in Tetherin sequence among primates, particularly at the target sites for Vpu and Nef, that the ancestors of modern viruses encountered as they were transmitted from species to species. Tetherin sequence diversity also means that the antagonists often exhibit species-specific activity. For example, SIV_MAC_ Nef antagonizes macaque (mac) Tetherin but is less active against African Green monkey (agm) Tetherin and inactive against human (hu) Tetherin, whereas SIVagmSab Nef antagonizes both macaque and agmTetherin but is not active against huTetherin [21]. A key determinant of sensitivity to Nef proteins is a five amino acid motif in the Tetherin CT [18], [21]; huTetherin is unique amongst primate Tetherins in that it lacks these five amino acids, and is resistant to all Nef proteins studied to date [20].

Nef is a 27–35 kD protein that is composed of (i) an N-terminal myristoyl membrane anchor, (ii) a flexible polypeptide chain that varies in length among Nef proteins, (iii) a polyproline helix type II that mediates interaction with SH3 domains (iv) a core domain that assumes a globular structure and is generally conserved among Nef proteins (iv) a C-terminal domain of unknown structure (reviewed in [25]). Nef proteins have been shown to downregulate several cell surface molecules, exploiting distinct cellular protein partners that interact with distinct Nef sequences for downregulation of various targets ([26], [27], reviewed in [28]). Thus, Nef recruits AP-1 to target MHC-I from the trans-Golgi to the lysosomes [29], [30] and induces MHC-I endocytosis from the plasma membrane [31]. Conversely, Nef promotes endocytosis of CD4 from the cell surface and a critical dileucine motif in Nef was shown to be required for both this activity and Nef binding to AP complexes [32], [33], [34], [35]. It was subsequently demonstrated that HIV-1 Nef employs two distinct motifs to form a complex with AP-2: an EXXXLL motif interacts with the AP-2 α-σ2 hemicomplex and a DD motif mediates additional interactions with AP-2 α and thus recruits AP-2 to mediate CD4 downregulation [36], [37]. Notably, both AP-2 interaction motifs are conserved in Nef proteins from a variety of SIVs.

The protein domains, cellular partners and mechanisms employed by Nef proteins to counteract Tetherin are unknown. Here we identify three key amino acid residues within SIVcpz Nef C-terminal flexible loop that are specifically required for Tetherin antagonism. We also demonstrate that Tetherin antagonism by Nef proteins from divergent SIVs is accompanied by the removal of Tetherin from sites of particle assembly and reduction of its levels at the cell surface, without effects on overall expression levels. We show that the two motifs in the Nef flexible C-terminal region that mediate interaction with AP-2 are also critical for these activities and, concordantly that AP-2 is required for Nef to antagonize Tetherin.

## Results

### Nef determinants specifically required for Tetherin antagonism

**Figure 1:**
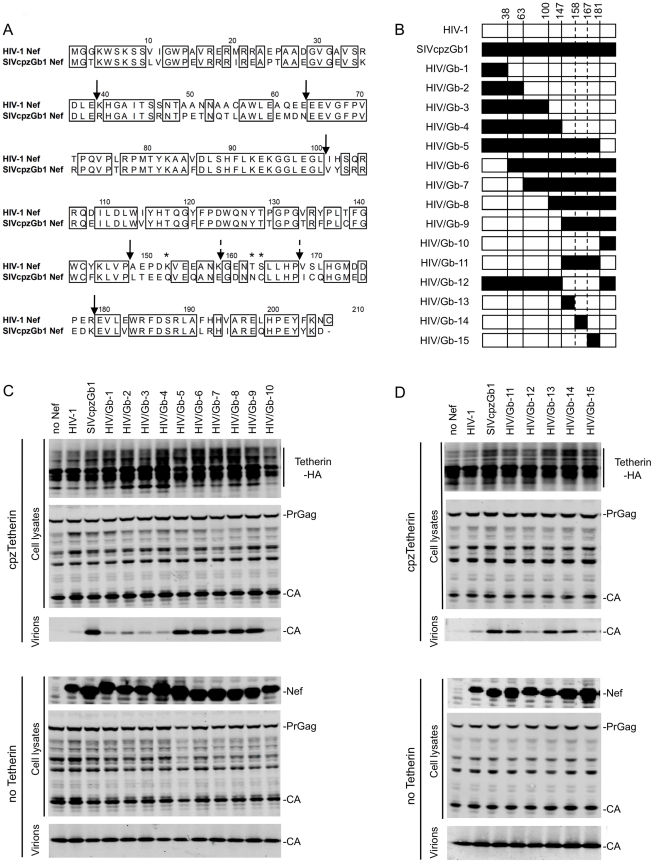
Mapping Tetherin antagonism determinants in Nef. (A) Amino acid alignment of HIV-1 (NL4.3) and SIVcpzGb1 Nef proteins. Identical residues are boxed. Arrows indicate the junctions at which chimeras between the proteins were generated. Stars indicate amino acids that were mutated in proteins used in Figure 2. (B) Schematic representation of chimeras generated between HIV-1 and SIVcpzGb1 Nef proteins. White boxes represent HIV-1 Nef-derived sequences and black boxes represent SIVcpzGb1 Nef-derived sequences. The amino acid number at which the junction between the two proteins was generated is indicated at the top of the diagram. (C and D) Quantitative fluorescence-based Western blot analysis of particle release. HIV-1 lacking Vpu and Nef was expressed together with the Nef proteins (*in trans*) indicated in the presence or absence of cpzTetherin. Cell and virion lysates were probed with an anti-capsid antibody. Cell lysates were also probed with anti-HA monoclonal antibody and an anti-HIV-1 Nef antibody that also recognizes SIVcpzGb1 Nef. The results shown are representative of 2-3 independent experiments. (See also Figure S1.).

We and others have previously shown that five amino acids in the Tetherin CT are necessary and sufficient to confer sensitivity to antagonism by Nef [18], [21]. To determine regions of Nef that are specifically required for Tetherin antagonism, we selected two Nef proteins that are closely related but differ in their ability to antagonize Tetherin. Specifically, SIVcpzGb1 Nef is a potent antagonist of cpzTetherin while HIV-1 Nef is completely inactive, even though the two proteins share ∼70% amino acid homology (Figure 1A). We generated a series of chimeric SIVcpz/HIV-1 Nef proteins, depicted in Figures 1A and 1B, and assayed their ability to antagonize cpzTetherin when co-expressed, *in trans*, with an HIV-1 construct lacking Vpu and Nef. Chimeric Nef protein expression was confirmed using western blot analyses with an antibody that recognizes both proteins (Figure 1C and 1D). Replacing increasingly larger portions of the N-terminal portion of the HIV-1 Nef protein by the corresponding regions from SIVcpzGb1 Nef gave rise to chimeras HIV/Gb-1 through -5. HIV/Gb-1,-2,-3 and -4, containing up to 147 N-terminal residues from SIVcpzGb1 Nef had the same phenotype as HIV-1 Nef in that they were unable to antagonize cpzTetherin. However, inclusion of the N-terminal 181 residues from SIVcpzGb1 Nef allowed the resulting HIV/Gb-5 chimera to antagonize cpzTetherin as efficiently as wild-type SIVcpzGb1 Nef (Figure 1C and Figure S1A). In a reciprocal set of chimeras, HIV/Gb-6 to HIV/Gb-10, SIVcpzGb1 Nef N-terminal sequences were replaced by those from HIV-1 Nef (Figure 1B). Chimeras HIV/Gb-6, -7, -8 and -9 that contained up to 147 N-terminal residues from HIV-1 Nef maintained cpzTetherin antagonism activity. However, replacement of 181 N-terminal residues by those from HIV-1 Nef, in the HIV/Gb-10 chimera, abolished its activity (Figure 1C and Figure S1A). There was only slight variation in the expression of these chimeric Nef proteins that did not correlate with their ability to counteract cpzTetherin (Figure 1C and 1D) and Nef expression levels were not affected by Tetherin expression (Figure S1B).

These data suggested that amino acids 148 to 181 of SIVcpzGb1 Nef contained key determinants of Tetherin antagonism. Thus, as expected, replacement of amino acids 148–181 in HIV-1 Nef by the corresponding residues from SIVcpzGb1 Nef resulted in chimera HIV/Gb-11 (Figures 1A and 1B) that was capable of antagonizing cpzTetherin restriction slightly less efficiently than wild-type SIVcpzGb1 Nef (Figure 1D and Figure S1C). The reciprocal chimera HIV/Gb-12, that contained HIV-1 Nef residues 148–181, in an otherwise SIVcpzGb1 background was unable to antagonize cpzTetherin. Subsequently, smaller portions of HIV-1 Nef within this region were substituted by the corresponding residues from SIVcpzGb1 Nef, generating chimeras HIV/Gb-13, -14 and -15 (Figure 1A and 1B). Chimera HIV/Gb-15 was unable to counteract cpzTetherin. In contrast both chimeras HIV/Gb-13 and -14 that encoded SIVcpzGab1 Nef residues 148–158 and 159–167, respectively, were able to antagonize cpzTetherin (Figure 1D and Figure S1C). Compared to wild-type SIVcpzGb1 Nef, the activity of these chimeras was somewhat reduced (Figure 1D and Figure S1C), suggesting that determinants within both regions 148–158 and 159–167 contribute to cpzTetherin antagonism. These chimeras were all expressed at comparable levels (Figure 1D and Figure S1D). Importantly, all of the aforementioned Nef chimeras maintained CD4 downregulation activity (Figure S1E), even though some chimeras exchanged Nef regions that have been shown to be required for CD4 downregulation [36], [37]. This result demonstrates that Nef functions other than Tetherin antagonism were not grossly affected in the chimeras.

**Figure 2:**
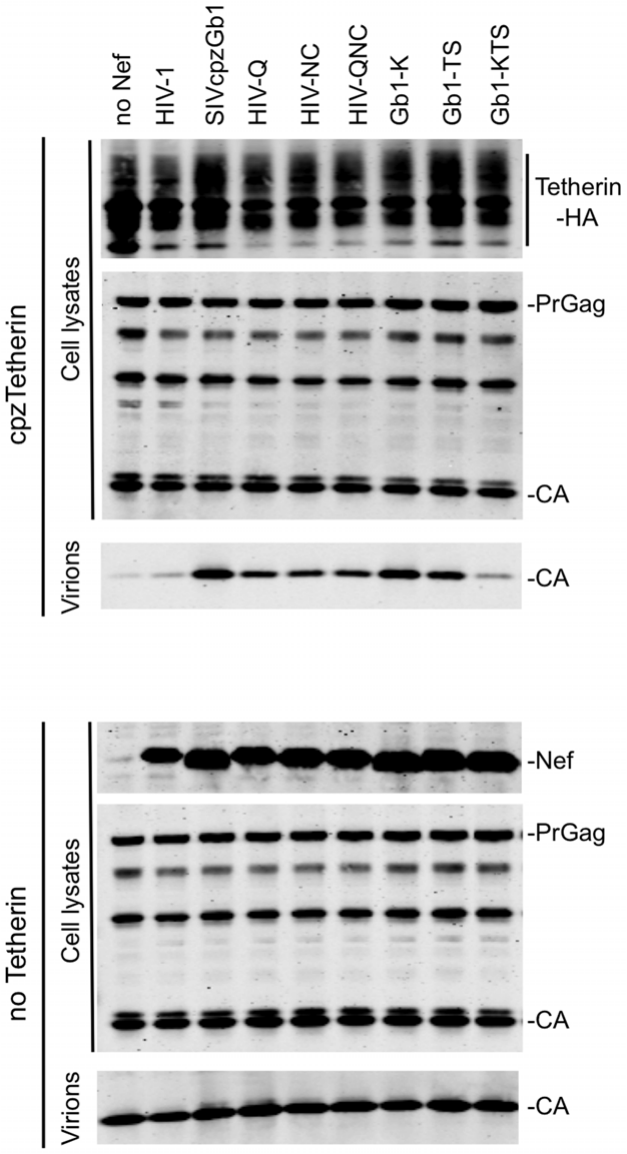
Identification of Nef amino acids important for Tetherin antagonism. Quantitative fluorescence-based Western blot analysis of particle release. HIV-1 lacking Vpu and Nef was expressed together with the Nef proteins (*in trans*) indicated in the presence or absence of cpzTetherin. Cell and virion lysates were probed with an anti-capsid antibody. Cell lysates were also probed with anti-HA monoclonal antibody and an anti-HIV-1 Nef antibody that also recognizes SIVcpzGb1 Nef. The results shown are representative of at least 3 independent experiments. (See also Figure S2.).

CpzTetherin antagonism is a conserved property of Nef proteins from SIVcpz strains and absent from all the HIV-1 M-group strains studied thus far [20]. In amino acid region 148–158 several amino acids differed between the HIV-1 and SIVcpz Nef proteins used here, but only one of these (K/Q at position 152) segregated according to whether the Nef proteins previously tested were from HIV-1 group M or SIVcpz strains (Figure 1A and [20]). In region 159–167, amino acids at positions 162 and 163 differed between HIV-1 Nef and SIVcpzGb1 Nef (Figure 1A), although their identity did not segregate perfectly between HIV-1 and SIVcpz Nef proteins [20]. Based on this analysis, we generated three mutant HIV-1 Nef proteins: 1) HIV-1Q, harboring one amino acid change: K152Q, 2) HIV-1NC, harboring two amino acid changes: T162N and S163C and 3) HIV-1QNC, where all three substitutions K152Q, T162N and S163C were made. Both the HIV-1Q and HIV-1NC mutants antagonized cpzTetherin, albeit with reduced efficiency compared to SIVcpzGb1 Nef (Figure 2 and Figure S2A). The combination of all three amino acid changes, in HIV-1QNC did not increase the efficiency of cpzTetherin antagonism over the HIV-1Q and HIV-1NC mutants suggesting that other residues within amino acid stretches 148–167 also contribute to anti-Tetherin activity. Protein expression levels were comparable for all mutant and wild type proteins (Figure 2 and Figure S2B).

In a reciprocal panel of SIVcpzGb1 mutants, introduction of the single mutation Q152K in SIVcpzGb1 Nef (Gb1-K) did not significantly affect its ability to inhibit cpzTetherin whereas a double mutation N162T and C163S (Gb1-TS) resulted in a very modest (2-fold) reduction in activity (Figure 2 and Figure S2A). However, when all three amino acid changes were combined, in Gb1-KTS, the resulting Nef protein was significantly impaired in its ability to antagonize cpzTetherin. The Gb1-KTS Nef protein retained its ability to downregulate CD4 although it was slightly less efficient than the parental and the other mutant Nef proteins (Figure S2C). These data indicate that residues Q152, N162 and C163 specifically affect the ability of SIVcpzGb1 Nef to counteract cpzTetherin, although other residues in the 148–167 domain also contribute to this activity.

### The AP-2 binding sites in SIV_MAC_, SIVagm and SIVcpz Nef proteins are required for Tetherin antagonism

**Figure 3:**
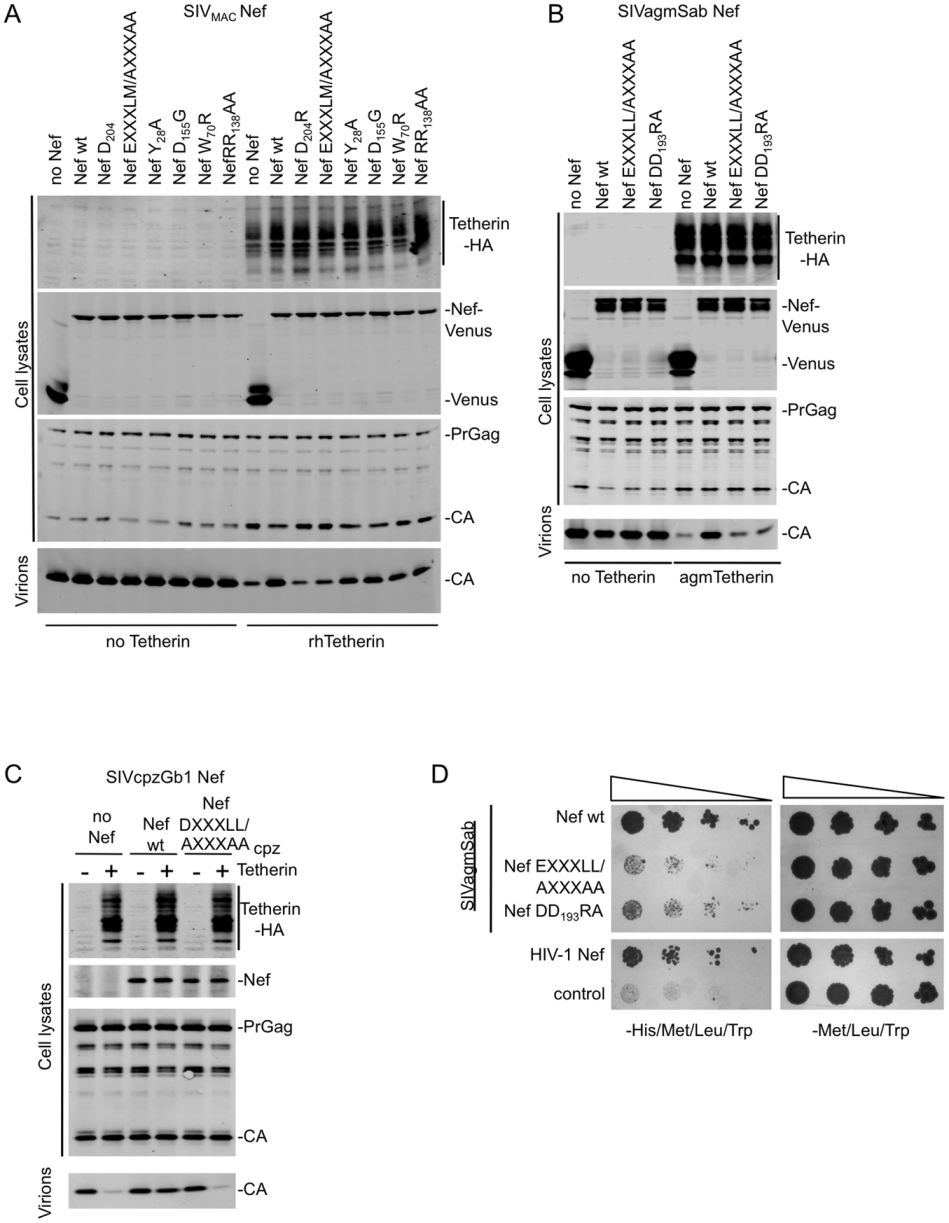
Mutation of Nef residues required for AP-2 binding abolish Tetherin antagonism. (A) Quantitative Western blot analysis of particle release for SIV_MAC_ based viruses lacking Nef, co-expressed with the indicated wild type or mutant SIV_MAC_ Nef proteins fused to Venus (*in trans*) in the presence or absence of rhTetherin. Cell and virion lysates were probed with an anti-HIV-1-capsid antibody. Cell lysates were also probed with anti-HA and anti-GFP monoclonal antibodies to determine rhTetherin and Nef-Venus protein expression levels respectively. The results shown are representative of at least 2 independent experiments. (B) Quantitative Western blot analysis of particle release for HIV-1 based viruses lacking Vpu and Nef, co-expressed with wild type or mutant SIVagmSab Nef proteins fused to Venus (*in trans*) in the presence or absence of agmTetherin. Cell and virion lysates were probed as in (A). The results shown are representative of at least 2 independent experiments. (C) Quantitative Western blot analysis of particle release for HIV-1 based viruses lacking Vpu and expressing either no Nef, or wild type or mutant SIVcpzGb1 Nef (*in cis*) in the presence or absence of cpzTetherin. Cell and virion lysates were probed with an anti-capsid antibody. Cell lysates were also probed with anti-HA monoclonal antibody and an anti-HIV-1 Nef antibody that also recognizes SIVcpzGb1 Nef. The results shown are representative of at least 2 independent experiments. (D) Y3H analysis of interaction between wild type and mutant SIVagmSab Nef and AP-2 α-σ2 hemicomplexes. Yeast cells were cotransformed with plasmids expressing the various indicated Nef proteins fused to a GAL4 binding domain and the AP-2 σ2 subunit and a plasmid expressing the AP-2 α subunit fused to a VP16 activation domain. Double transformants were selected on dropout plates lacking Leu, Trp and Met. Pooled colonies from each plate were normalized for cell number and serial dilutions plated on plates lacking Leu, Trp and Met as a control and plates lacking Leu, Trp, Met and His to detect protein-protein interaction. The results shown are representative of 3 independent experiments. (See also Figure S3).

We have previously shown that a mutation in SIV_MAC_ Nef (D_204_R) that inhibits CD4 downregulation also reduces its ability to counteract rhTetherin while a mutation that abolishes MHC-I downregulation (Y_223_F) does not affect Tetherin antagonism [21]. Because MHC-I downregulation by Nef requires AP-1 [30] while CD4 downregulation is mediated through AP-2 recruitment [36], it seemed plausible that Nef might recruit AP-2 to remove Tetherin from the cell surface and rescue virus release. Two motifs in the HIV-1 Nef C-terminal flexible loop are required for interaction with AP-2, namely EXXXLL_165_ and DD_175_ (Figure 1A) [36], [37]. SIV_MAC_ Nef proteins bearing mutations at the corresponding motifs (EEHYLM_195_ to AEHYAA and D_204_ to R) were expressed as fusion proteins with the fluorescent protein Venus at the C-terminus. The wild type SIV_MAC_Nef-Venus fusion protein efficiently antagonized rhTetherin, but as we have previously shown [21], the D_204_R mutation abolished this activity (Figure 3A and Figure S3A). Similarly, mutation of the leucine-based motif (EXXXLM_195_/AXXXAA) in SIV_MAC_ Nef that mediates interaction with the AP-2 α-σ2 hemicomplex [36] also abolished rhTetherin antagonism.

To determine potential roles in Tetherin antagonism of a number of SIV_MAC_ Nef residues, previously reported to mediate interaction with several cellular proteins, we tested Nef proteins that were mutated at the following positions: 1) Y_28_ (to A) that has been suggested to affect interactions with AP-1 and AP-2 proteins [38], [39], 2) W_70_ (to R) that corresponds to residue W_57_ in HIV-1 Nef shown to be involved in CD4 binding [40], 3) D_155_ (to G) that affects Nef dimerization [41] and 4) R_138_R_139_ (to AA) that affects Pak1/2 binding [42]. None of the above mutations had major effects (<2–3-fold) on particle release in experiments where SIV_MAC_Nef was asked to antagonize rhTetherin (Figure 3A and Figure S3A). These data suggest that mutations that impair SIV_MAC_ Nef interactions with other proteins do not generally affect its ability to antagonize rhTetherin. The notable exceptions were mutations that disrupt the leucine-based and diacidic motifs involved in AP-2 binding.

These two AP-2-binding motifs are highly conserved among lentiviral Nef proteins and we therefore asked whether their mutation in other Nef proteins from SIVagm and SIVcpzGb1 that antagonize Tetherin, would also affect their activity. Indeed, mutation of either AP-2-binding motif in SIVagmSab Nef, EXXXLL_183_ or DD_193_, significantly reduced the ability of SIVagmSab Nef to rescue particle release from inhibition by agmTetherin (Figure 3B and Figure S3B). Similarly, mutation of the DXXXLL_165_ motif in SIVcpzGb1 Nef completely abolished its ability to counteract cpzTetherin (Figure 3C and Figure S3C).

AP-2 has been previously shown to interact with HIV-1 and SIV_MAC_ Nef [36], [38], but not with SIVagmSab or SIVcpzGb1 Nef. We used a previously described yeast-3-hybrid assay to test for such an interaction [36], [37]. The Nef proteins were fused to the Gal4 binding domain in a vector also expressing the AP-2 σ2 subunit and tested for interaction with a vector expressing the AP-2 α subunit and the VP16 activation domain. Like HIV-1 Nef, SIVagmSab Nef interacted with AP-2 α-σ2 hemicomplex, and mutation of either the EXXXLL_183_ motif or DD_193_ abolished this interaction (Figure 3D). Unfortunately, in our hands both SIV_MAC_ and SIVcpzGb1 Nef proteins induced activation of transcription even in the absence of AP-2 subunits and thus could not be tested in this assay. Therefore, we used an alternative assay to determine whether the Nef-Venus fusion proteins co-localized with endogenous AP-2 in mammalian cells. Indeed all three proteins SIV_MAC_, SIVagmSab and SIVcpzGb1 Nef showed a clear and obvious co-localization with AP-2 at the cell surface (Figure S4). Altogether, this data suggested that Nef residues that are important for interaction with AP-2 were necessary for Nef proteins to efficiently antagonize Tetherin.

### AP-2 binding sites in Nef are required for Tetherin downregulation from the cell surface

Since Nef recruits AP-2 to downregulate CD4 from the cell surface we determined whether Nef could downregulate Tetherin and whether the AP-2 binding sites were required for this function. Because we wished to analyze Tetherins from various species, but only antibodies that recognize the extracellular domain of mouse (mo) Tetherin that work well in flow cytometric assays are commercially available, we generated a panel of chimeric Tetherins. These contained the extracellular domain of moTetherin linked to the transmembane (TM) and CT domains from (i) huTetherin, (ii) a modified human Tetherin, termed hu(GDIWK), in which 5 amino acids that confer sensitivity to antagonism by SIV_MAC_ and SIVagm Nef [21] were reintroduced into the huTetherin CT and (iii) cpzTetherin (Figure S5A). Notably the structure of moTeherin and huTetherin extracellular domains are nearly super-imposable [6]–[8] and clones of 293T cells stably expressing these Tetherins exhibited the expected phenotype in that they inhibited release of HIV-1 virions lacking Vpu and Nef, but expression of the appropriate Nef protein rescued particle release (Figure S5B and C).

**Figure 4:**
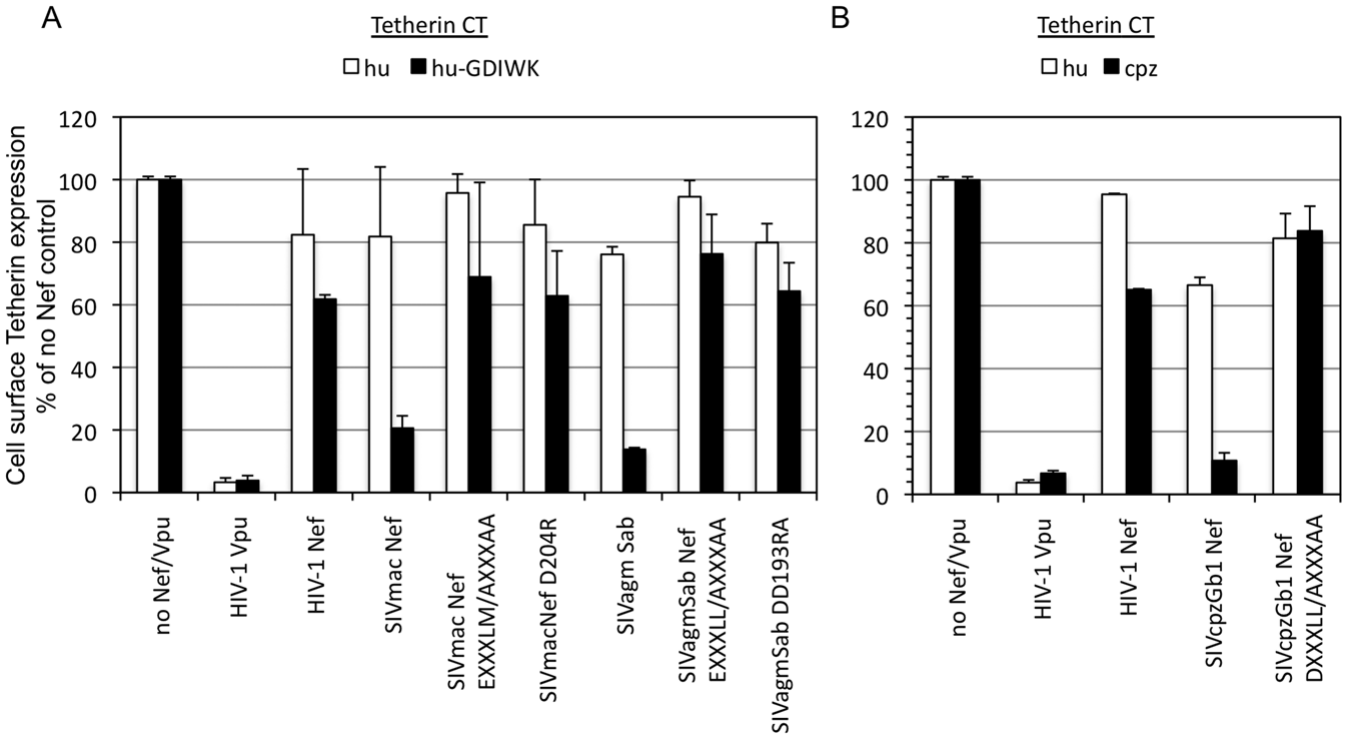
Mutation of Nef residues involved in AP-2 binding affects cell surface downregulation of Tetherin. (A) Surface staining of cells stably expressing chimeric mo-Tetherin containing the hu-Tetherin CT unmodified or with a 5 amino acid (GDIWK) insertion. Cells were transduced with HIV-based vectors expressing the indicated Nef proteins or HIV-1 Vpu and IRES-GFP and stained with anti-mouse Tetherin antibody conjugated to APC. The median fluorescence in the APC channel of GFP positive cells is plotted as a proportion of the median fluorescence of cells transduced with a control empty vector which was set at 100%. Data is plotted as the mean and standard deviation of 3 independent experiments. (B) Surface staining of cells stably expressing chimeric mo-Tetherin containing the hu- or cpz-Tetherin CT. Cells were transduced stained and fluorescence quantitated as in (A). (See Figures S5 and S6).

The various Tetherin-expressing cell lines were transduced with HIV-1 based vectors expressing Nef-IRES-GFP cassettes and Tetherin cell surface expression was assessed by FACS using an antibody that recognizes the extracellular region of moTetherin. HIV-1 Vpu was used as a control in place of Nef; because it targets the TM domain of Tetherin which is well conserved between cpzTetherin and huTetherin, and caused downregulation of all the chimeric Tetherin proteins used (Figure 4A and 4B). In agreement with previously published data [18], we observed modest downregulation of the hu(GDIWK) but not the huTetherin chimera by SIV_MAC_ Nef (Figure 4A and Figure S6A). Notably, mutations of either the EXXXLM_195_ or D_204_ residues in SIV_MAC_ Nef abolished this activity. The effects of SIVagmSab Nef on cell surface hu(GDIWK) Tetherin expression were more pronounced (Figures 4A and Figure S6A) and again downregulation was abolished by mutations in either the EXXXLL_183_ or DD_193_ motifs (Figures 4A and Figure S6A). Similarly, SIVcpzGb1 Nef significantly decreased cell surface expression of the cpzTetherin chimera and mutation of the DXXXLL_165_ motif abolished this ability (Figures 4B and Figure S6B). Importantly, the effects on Tetherin downregulation were specific, because none of the Nef proteins affected cell surface expression of the huTetherin chimera (Figure 4A and 4B and Figure S6A and S6B). Thus the AP-2 binding side in three widely divergent Nef proteins was required for Tetherin downregulation from the cell surface.

To determine whether Nef affects Tetherin endocytosis, cells expressing either chimeric hu-moTetherin or cpz-moTetherin proteins were transduced with vectors expressing no Nef or wild type SIVcpzGb1 Nef or the SIVcpzGb1 Nef DXXXLL_165_ (AXXXAA) mutant. Cells were incubated in the cold with a fluorescently labeled anti-mouse Tetherin antibody, washed, and then shifted to 37°C. The fraction of Tetherin that was internalized at various times after the temperature shift was then determined based on the amount of fluorescence that became resistant to an acid wash. These data suggested that a fraction (about half) of the Tetherin was rapidly and constitutively endocytosed within a few minutes, while the remainder was present at the cell surface for >40 minutes (Figure S7). Wild type SIVcpzGb1 Nef but not the DXXXLL_165_ mutant increased the fraction of cpz-moTetherin that was rapidly internalized within 10 min of the temperature shift to ∼100% but did not affect hu-moTetherin endocytosis (Figure S7). This data suggests that Nef downregulates Tetherin from the cell surface by increasing the amount of Tetherin that undergoes rapid endocytosis, to include the majority of cell surface molecules and that this activity is dependent on the Nef AP-2 binding site. Alternatively, this data is also compatible with the hypothesis that a pool of Tetherin is constitutively rapidly internalized and recycled to the cell surface, and that Nef interferes with this cycle by causing its entrapment at intracellular locations.

### Nef removes Tetherin from sites of particle assembly

**Figure 5:**
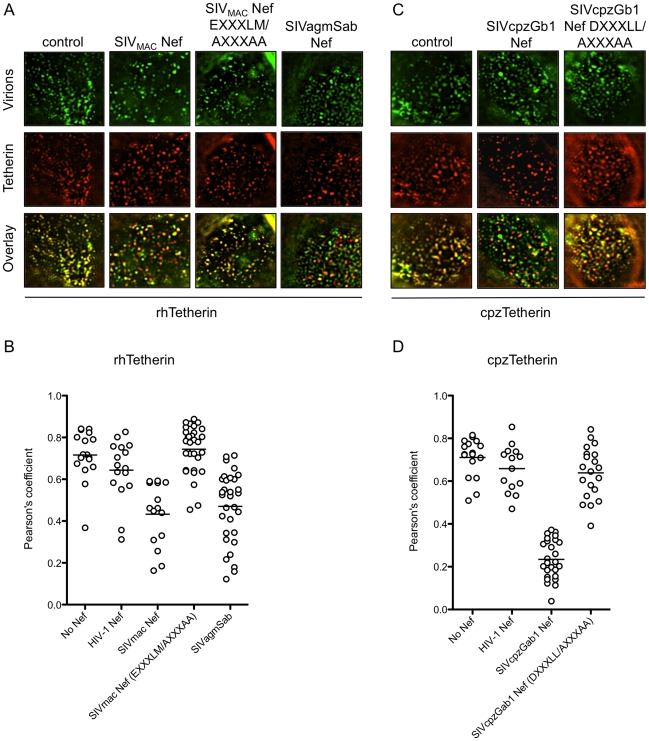
AP-2 binding sites in Nef are required to remove Tetherin from sites of particle assembly. (A) Immunofluorescence analysis of cells stably expressing HA-tagged rhTetherin and transfected with HIV-1 proviral plasmids expressing YFP-labelled Gag and various Nef proteins (for details see Materials and Methods). Tetherin appears red and viral particles appear green. Representative images of the apical portion of a cell are shown for each Nef protein. (B) Quantitative analysis of co-localization between rhTetherin and YFP-labeled HIV-1 particles in the presence of the indicated Nef proteins. Each symbol represents the value of the Pearson's coefficient for an individual cell and the horizontal line represents the mean value for 15 to 30 cells. (C) As in (A) but cells expressed HA-tagged cpzTetherin. (D) Quantitative analysis of co-localization between cpzTetherin and YFP-labeled viral particles, as in (B).

In the presence of huTetherin and absence of HIV-1 Vpu, fluorescently labeled nascent HIV-1 particles are seen trapped on the cell surface and colocalizing with huTetherin ([12] and unpublished data). To determine whether Nef proteins inhibited colocalization of Tetherin with nascent virions, YFP-labelled HIV-1 particles were generated in cells stably expressing HA-tagged rh- or cpzTetherin in the absence or presence of wild-type and mutant Nef proteins, and surface Tetherin protein revealed by immunostaining of non-permeabilized cells. In absence of Nef, nascent HIV-1 particles exhibited strong co-localization with both rhTetherin and cpzTetherin (Figure 5A–D). Coexpression of HIV-1 Nef had no effect on this colocalization, but SIV_MAC_ and SIVagmSab Nef proteins significantly inhibited the colocalization between viral particles and rhTetherin (Figure 5A and 5B). Similar, but more pronounced effects were seen with SIVcpzGb1 Nef, which disrupted the co-localization between HIV-1 particles and cpzTetherin (Figure 5C and 5D). For both SIV_MAC_ and SIVcpzGb1 Nef proteins, mutation of the E/DXXXLM/L motif abolished their ability to remove their respective Tetherin targets from sites of particle assembly (Figure 5A–D). Thus, Nef proteins removed Tetherins from sites of particle assembly and this activity was dependent on motifs critical for Nef-AP-2 interaction.

### AP-2 depletion inhibits the ability of Nef to antagonize Tetherin

**Figure 6:**
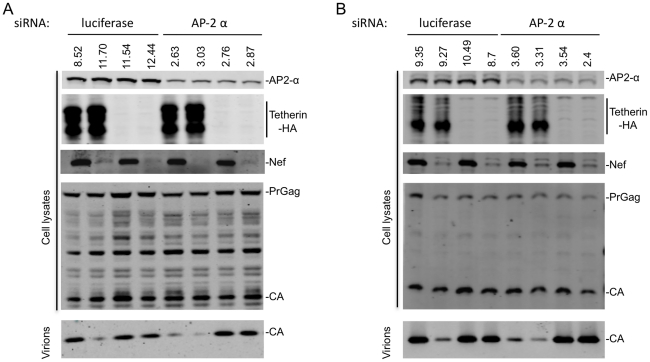
AP-2 is required for Nef to antagonize Tetherin. (A) Quantitative Western blot analysis of particle release from cells transfected with the indicated siRNA, an HIV-1 provirus lacking Vpu (and expressing no Nef or SIVcpzGb1 Nef) and cpzTetherin or a control plasmid. Cell and virion lysates were probed with an anti-capsid antibody. Cell lysates were also probed with anti-Nef, anti-HA and anti-AP-2α antibodies. Numbers above the AP-2 α blot represent measurement of AP-2 α protein expression levels (LICOR). A representative of 2 independent experiments is shown. (B) Same as (A) but particle release by SIV_MAC_ or SIV_MAC_ΔNef in the presence or absence of rhTetherin is shown. A representative of 2 independent experiments is shown.

To demonstrate that AP-2 is important for Tetherin antagonism by Nef, we used siRNA based approaches to deplete the α subunit of AP-2, since Nef binds directly to this subunit [36], [37], [43]. Transfection of 293T cells with AP-2 α-specific siRNAs reduced AP-2 α subunit expression levels by ∼70–80% (Figure 6A and 6B). AP-2 α depletion did not affect particle release in the absence of Tetherin, nor did it affect the ability of Tetherin to inhibit particle release in the absence of Nef. However, the ability of SIVcpzGb1 Nef to rescue particle release from inhibition by cpzTetherin was greatly attenuated when AP-2 α was depleted (Figure 6A and Figure S8A). Similarly, AP-2 α depletion resulted in nearly complete loss of the ability of SIV_MAC_ Nef to antagonize rhTetherin (Figure 6B and Figure S8B). Thus, endogenously expressed AP-2 α is required for Nef proteins to inhibit Tetherin.

**Figure 7:**
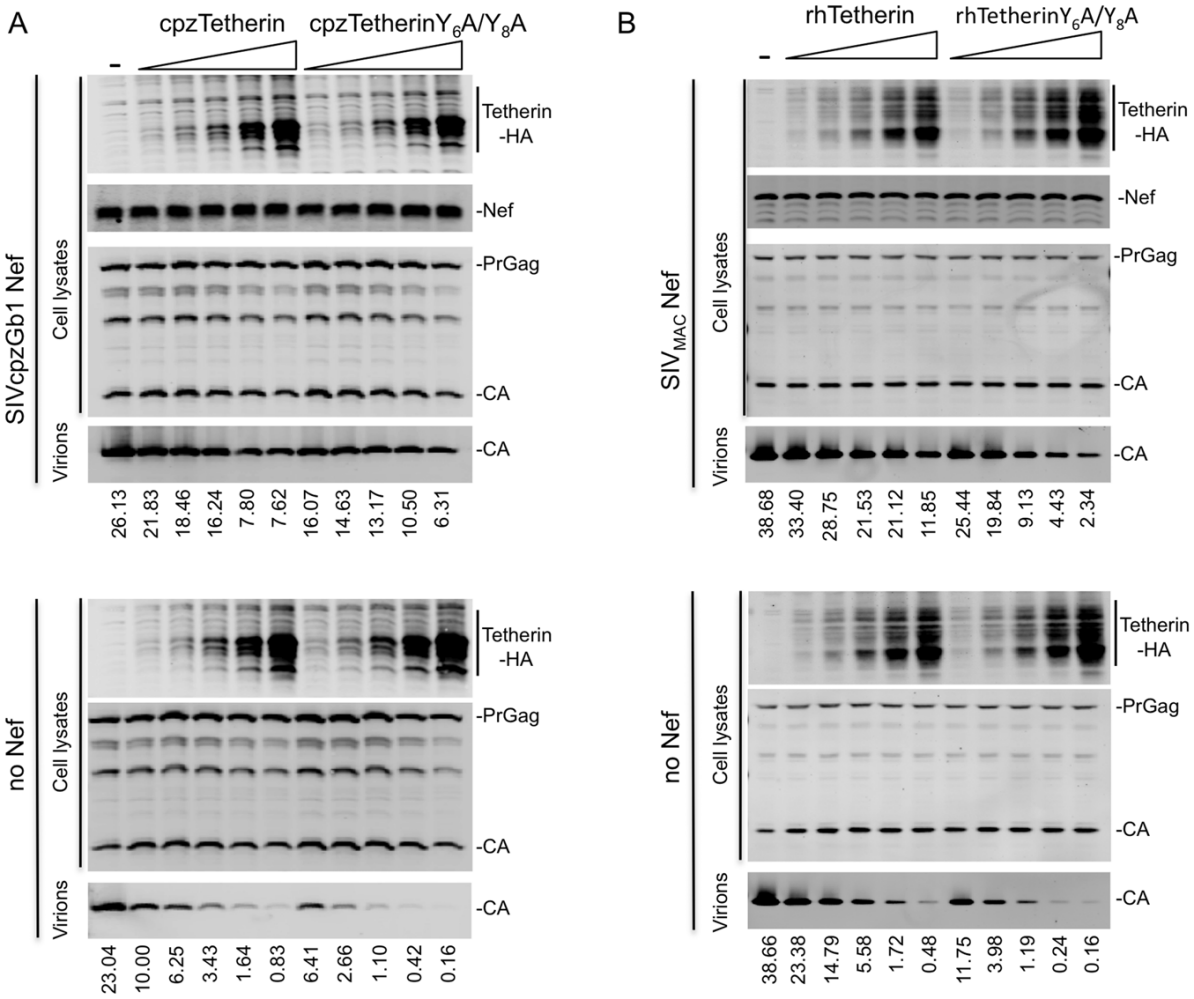
Direct binding of Tetherin to AP-2 is dispensable for Tetherin function and antagonism. (A) Quantitative Western blot analysis of particle release from cells expressing an HIV-1 provirus lacking Vpu and expressing no Nef or SIVcpzGb1 Nef in the absence or presence of increasing amounts of unmodified or Y6A/Y8A mutant cpzTetherin. Cell and virion lysates were probed with anti-capsid and anti-HA antibodies. Numbers at the bottom indicate measurement of viral particle release (LICOR). A representative of 2 independent experiments is shown. (B) Quantitative Western blot analysis of particle release from cells expressing SIV_MAC239_ or SIV_MAC239_ΔNef in the absence or presence of increasing amounts of wild type or Y6A/Y8A mutant rhTetherin. Cell and virion lysates were probed as in (A). A representative of 2 independent experiments is shown. (See Figure S9.).

Curiously, Tetherin proteins themselves contain a YXXV/M/I motif in their CT that could mediate binding to AP-2, specifically the μ2 subunit [44]. Indeed rat Tetherin has been shown to bind to μ2 and to be subject to AP-2 mediated endocytosis [45]. huTetherin has also been reported to undergo AP-2-dependent endocytosis, and two tyrosine residues (Y6 and Y8) in the huTetherin CT were found to be important for AP-2 α subunit binding [46]. To determine whether these residues were important for Tetherin activity, or for sensitivity to Nef, we mutated both tyrosines in cpz- and rh-Tetherin. Increasing amounts of unmodified or mutant cpzTetherin were co-expressed with a Vpu-defective HIV-1 virus that expressed either no Nef or SIVcpzGb1 Nef. In the absence of Nef, viral particle release was inhibited to similar extents by both the mutant and the unmodified Tetherin proteins (Figure 7C). Similar results were obtained when increasing amounts of unmodified or mutant rhTetherin were co-expressed with SIV_MAC_ or SIV_MAC_ΔNef (Figure 7D). Importantly, for both cpzTetherin/SIVcpzGb1 Nef and rhTetherin/SIV_MAC_ Nef, the presence or absence of the reported AP-2 binding site in the Tetherin CT did not affect sensitivity to antagonism by the corresponding Nef protein (Figure 7C and 7D). Furthermore, cpzTetherin downregulation by SIVcpzGb1 Nef was also unaffected by mutation of the cpzTetherin AP-2 binding site (Figure S9). Thus, interactions between tyrosine motif in the Tetherin CT and AP-2 are not required for Tetherin activity, or sensitivity to Nef proteins. Rather, interactions between Nef and AP-2 are key for its Tetherin antagonist function.

## Discussion

Here, we show that Nef antagonizes Tetherin by decreasing its levels at the cell surface and particularly at sites of particle assembly. We also show that the ability of Nef to antagonize Tetherin is lost when residues that are important for AP-2 binding are mutated, or when AP-2 α expression is reduced using RNA interference. These results strongly suggest that the interaction of Nef with AP-2 is required for the removal of Tetherin from sites of virion assembly and antagonism of its antiviral function. Additionally, we identified three amino acids at the C-terminal loop of SIVcpzGb1 Nef, that are key determinants of its ability to antagonize Tetherin. However, our data suggest that other residues within the C-terminal loop and particularly within amino acids 148 to 167 also contribute to Tetherin antagonism. Together, these findings suggest a model in which Nef interacts with Tetherin and simultaneously with AP-2 via the Nef C-terminal loop and that the formation of this complex impairs the ability of Tetherin to be incorporated into virions, because it becomes sequestered by Nef and AP-2 away from sites of particle release, and either internalized or trapped at intracellular locations more efficiently than it would otherwise be. Interestingly, the decreased levels of Tetherin at the cell surface in the presence of Nef do not lead to an obvious reduction in the total Tetherin protein levels in the cell (Figure 3 and Figure 4 and data not shown). This data suggests that in contrast to CD4 [32], Tetherin internalization or intracellular retention by Nef-AP-2 does not lead to Tetherin degradation.

The reciprocal specificity associated with the sequence requirements in both Nef and Tetherin for antagonism are most consistent with the notion that the two proteins directly interact with each other. Unfortunately, we were not able to demonstrate a specific physical interaction between Nef and Tetherin using *in vitro* binding assays with recombinant proteins, yeast two-hybrid assays, or co-immunoprecipitation experiments in mammalian cells. This suggests that the putative Nef-Tetherin interaction (if it occurs) is of low affinity or unstable outside the confines of a cell membrane. Interestingly, the Nef C-terminal flexible loop, in which key determinants of Tetherin antagonism reside, also mediates interaction with the AP-2 complex. In fact, two key amino acids identified in SIVcpzGb1 Nef as important for Tetherin antagonism (T162 and C163) are embedded within the D/EXXXLL motif that is critical for interaction with the AP-2 α subunit (specifically within the sequence DNNCLL). Mutations at these variable positions in the otherwise very well conserved D/EXXXLL motif do not grossly affect the ability of Nef to downregulate CD4 (Figure S1), indicating that AP-2 binding and Tetherin antagonism are separable activities. Nonetheless, the AP-2 binding site and residues required for Tetherin antagonism are in close physical proximity. It is also noteworthy that the cytoplasmic tails of primate Tetherins contain a classical sorting signal recognized by the AP-2 μ subunit [44]. Indeed, rat Tetherin has been shown to interact with the AP-2 μ subunit [45] but huTetherin endocytosis has been reported to be dependent on the AP-2 α subunit and to be dependent on two tyrosine residues, one of which is part of the YXXV motif [46]. Importantly, however, although Tetherin may interact directly with AP-2, our findings indicate that this interaction is not required for virus restriction nor for sensitivity to Nef. This is in contrast with a recent report that demonstrates that the tyrosine residues in rhTetherin are critical for antagonism by an adapted SIVmac239 Env protein that causes rhTetherin downregulation, although the role of AP-2 in this activity was not determined [47].

Given the close proximity of amino acids putatively involved in Tetherin recognition to the AP-2 binding site, it might appear difficult to envisage how Nef might simultaneously bind to Tetherin and AP-2. It is important to note however, that residues interspersed with those comprising the AP-2 binding site (within the D/EXXXLL motif) in Nef likely form only part of the determinant for Tetherin recognition. Indeed, mutation of the two residues alone within the D/EXXXLL motif that are important for Tetherin antagonism, is not sufficient to abolish the Tetherin antagonizing activity of SIVcpzGb1 Nef. Additionally, introduction of the three residues Q152/N162/C163 (that include N162/C163 within the DXXXLL motif) identified as critical for Tetherin antagonism in SIVcpzGb1 Nef to HIV-1 Nef was not sufficient to confer anti-Tetherin activity at the levels obtained with wild type SIVcpzGb1 Nef. Interestingly, residues N162/C163 are naturally found in a number of HIV-1 Nef proteins, including in Nef from strains JR-CSF and YU-10x that have been previously shown to have no activity against cpzTetherin [20]. Therefore, the context within which Nef residues are mutated determines whether they confer anti-Tetherin activity and several different residues within the C-terminal flexible loop of Nef contribute to Tetherin antagonism. It is possible that Nef forms multiple (perhaps individually weak) contacts with Tetherin, only one of which is mediated by an overlapping motif with the Nef-AP-2 binding site. One possible model, that is consistent with our findings, is that binding of the flexible C-terminal loop of Nef to AP-2 results in the formation of the site on Nef that binds to Tetherin. It is even possible that both AP-2 and Nef residues contribute to the formation of the Tetherin binding site. Alternatively, it has been reported that Nef can form dimers [41], so it is possible that one molecule in a Nef dimer interacts with Tetherin and the other with AP-2. However, arguing against this notion is our finding that mutation of a residue predicted to be required for SIV_MAC_ Nef dimerization did not affect its ability to counteract rhTetherin (Figure 3A). Finally, we cannot exclude the possibility that other cellular proteins, including other AP complexes, act as a bridge between the Nef-AP-2 complex and Tetherin, although this possibility seems unlikely due to the sequence specificity associated with the Nef-Tetherin antagonism.

Of the known accessory proteins encoded by lentiviruses, none has been ascribed more functions than Nef (reviewed in [28]). Nef is critical for efficient replication *in vivo* [48]–[50] and at least some of its reported functions, e.g. CD4 and MHC-I downregulation, are likely to play a significant role in pathogenesis. While it remains to be proven that Tetherin antagonism is required for efficient replication and dissemination of enveloped viruses *in vivo*, the finding that primate lentiviruses have evolved diverse strategies to counteract this antiviral protein suggests that it is. The identification of Nef domains and sequences that are required for Tetherin antagonism but not for other Nef activities could potentially allow the design of experiments to determine the importance of this function in viral pathogenesis *in vivo*.

We note that Nef antagonizes Tetherin via a mechanism that is related to that which it employs to downregulate CD4, namely recruitment of the AP-2 complex. Thus, it appears that Nef can parasitize the AP-2 complex for multiple ends. In a sense, the acquisition of Tetherin antagonism activity appears to be the result of the modification of an existing activity, namely CD4 downregulation, by broadening the array of target proteins to which AP-2 is recruited to include Tetherin. A similar concept might apply in the evolution of Vpu function, where (again) CD4 downregulation and Tetherin antagonism may proceed by similar mechanisms, with the viral accessory protein acting to recruit cellular factors (in that case β-TRCP [51]–[53]) to remove two different cellular molecules. Thus, lentiviruses can exploit Nef, and perhaps other accessory gene products, as somewhat plastic adaptors to recruit a given protein complex, in this case AP-2, to diverse targets so as to manipulate host cells to provide a more permissive environment for virus replication and dissemination.

## Materials and Methods

### Plasmid construction

Plasmids expressing wild-type HIV-1 and SIVcpz Gb1 Nef proteins have been previously described [20]. Chimeric proteins were made by overlap-extension PCR using external primers introducing XbaI and MluI sites at the 5′end and 3′end of the Nef coding sequence, respectively and internal primers. PCR products were cloned into pCG-IRESGFP [21]. The HIV-1-based proviral plasmids, pBRHIV-1NL4-3ΔVpu, lacking Vpu and expressing wild-type versions of Nef proteins from HIV-1, SIVcpz Gab, SIV_MAC239_, or SIVagmSab Nef *in cis*, have been previously described [20]. Mutations in the Nef coding sequences of these plasmids were generated using overlap-extension PCR and BamHI (in the Env coding region) and MluI (at the 3′-end of the Nef coding region) sites in pBRHIV-1NL4-3ΔVpu plasmid. The same strategy was used to introduce wild type and mutant Nef proteins into a pBRHIV-1NL4-3ΔVpuΔNef plamids bearing YFP embedded in sequences encoding the stalk region of matrix (MA). The pV1-derived plasmids encoding full length SIV_MAC23_ or SIV_MAC239_ΔNef proviruses have been previously described [21]. pCR3.1 plasmids were used to express wild-type and mutant SIV_MAC239_ and SIVagmSab Nef proteins fused to Venus at their C-terminus *in trans*. To express Nef proteins in 293T cell lines stably expressing Tetherin, Nef-IRES-GFP containing regions from pCG derivatives were transferred using SnaBI-NotI fragments (encompassing the CMV promoter-Nef-IRES-GFP cassette) into pCCGW, a HIV-based retroviral vector derived from pHRSIN-CSGW [54] by replacing the SSFV promoter with that from CMV. Plasmids expressing HA-tagged Tetherin proteins were constructed as previously described [12], [55]. Mutations of the tyrosine residues in the CT of rh- and cpzTetherin were introduced by overlap-extension PCR. Chimeric hu-mo or hu-GDIWK-mo or cpz-mo Tetherin proteins, were generated by overlap-extension PCR, using hu, hu-GDIWK [21] or cpzTetherin as template for the CT and TM domains and moTetherin as template for the extracellular domain. PCR products were inserted into a retroviral vector pLHCX (Clontech). All cloned coding sequences were verified by DNA sequencing. Oligonucleotide sequences are available upon request.

### Transfections and FACS analysis

293T and TZMbl cells were maintained under standard conditions. Transfection protocols have been previously described [21]. Several 293T-derived cell lines stably expressing HA-tagged rh- or cpzTetherin and chimeric hu-mo, hu-GDIWK-mo and cpz-mo Tetherin were derived by transduction of 293T cells with the corresponding retroviral plasmids, followed by selection in hygromycin (5 µg/ml).

To determine the ability of Nef proteins to counteract Tetherin, cells were transfected with 100-200 ng of pCG-IRES-GFP derived plasmids expressing various Nef proteins, 400–500 ng pBRHIV-1NL4-3ΔVpuΔNef or SIV_MAC_ΔNef and 20–25 ng of pCR3.1 Tetherin-HA expression plasmids or empty expression vector.

To test the activity of tyrosine mutant rh- and cpzTetherin proteins, transfections were performed as above but with increasing amounts (0 ng, 5 ng, 10 ng, 20 ng, 40 ng, 80 ng/well) of each Tetherin-HA expression plasmid. The total amount of DNA was held constant by supplementing the transfection with empty expression vector.

To quantify Tetherin downregulation by Nef, viral stocks were generated by transfecting 5μg of pCCGW plasmids expressing various Nef proteins or HIV-1 Vpu, 5μg of an HIV-1 Gag-Pol expression plasmid and 1μg of VSV-G expression plasmid in 293T cells (10 cm dishes) and used to inoculate cells stably expressing chimeric moTetherin proteins. At 48 h post-infection, cells were detached from plates with 5 mM EDTA in PBS and stained for cell surface Tetherin expression with anti-moTetherin antibody conjugated to APC (anti-mPDCA-1-APC, Miltenyi Biotec). The amount of cell-associated APC and GFP fluorescence was measured with an LSRII flow cytometer (BD).

### Virus release and immunoblot assays

Experiments were performed as previously described [21]. Immunoblots were probed with the following antibodies: rabbit anti-HA antibody (Rockland) or mouse anti-AP2 α (α-Adaptin 1/2, Santa Cruz Biotech.), mouse anti-HIV-1-p24CA (183-H12-5C), mouse anti-HIV-1-Nef (1539) or mouse anti-SIV_MAC_-Nef (17.2) (2659) (from the NIH AIDS Research and Reagents Program),followed by anti-rabbit or anti-mouse antibodies conjugated to IRDye680 or IRDye800 CW and scanned with an Odyssey Infrared Imager (LICOR).

### Microscopy

Cells stably expressing HA-tagged cpzTetherin or rhTetherin were seeded on 3.5-cm, glass-bottomed dishes coated with poly-L-Lysine (Mattek). Cells were transfected with 150 ng of an HIV-1 proviral plasmid (pBRHIV-1NL4-3ΔVpuΔNef) that was engineered to express various Nef proteins and 150 ng of an identical construct that expressed YFP embedded within the MA domain of Gag (pBRHIV-1NL4-3ΔVpuΔNef-MA(YFP)) using PEI (PolySciences). At 48 h post-transfection, cells were fixed with 4% paraformaldehyde and incubated with mouse anti-HA.11 monoclonal antibody (Covance) followed by anti-mouse IgG Alexafluor-594 conjugate (Molecular Probes). A Z-series of images was captured from the top apical half of the cells using an Olympus IX70-based Deltavision microscope and were then deconvolved with SoftWorx software (Applied Precision). For each cell, colocalization of Tetherin with virions was measured by tracing regions of interest in each Z-slice so as to analyze only the cell surface, and the Pearson's correlation coefficient for colocalization for each individual cell was calculated using SoftWorx software.

For the Nef-AP-2 co-localization assays, 293T cells, seeded on 3.5-cm glass-bottomed dishes coated with poly-L-Lysine (Mattek), were transfected with 100 ng of plasmids expressing Nef proteins fused to Venus at their C-terminus. At 48 h post-transfection, cells were fixed with 4% paraformaldehyde, permeabilized with 0.1% Triton and incubated with mouse anti-AP-2 α momoclonal antibody (Santa Cruz Biotech. α-Adaptin 1/2) followed by anti-mouse IgG Alexa Fluor-594 conjugate (Molecular Probes). A Z-series of images was acquired using Deltavision microscope, and colocalization between Nef and AP-2 was inspected using images of the cell surface acquired at the cell coverslip interface.

### Tetherin internalization assays

The internalization of tetherin from cell surface was determined by flow cytometry assay as described previously for other cell surface markers [56], [57]. Briefly, cells stably expressing chimeric moTetherin proteins were transduced with pCCGW Nef-IRES-GFP vectors plasmids. At 40 h post-infection, 10^7^ cells were incubated with saturating amounts of anti-moTetherin antibody conjugated to APC (anti-mPDCA-1-APC, Miltenyi Biotec) in DMEM containing 0.5% bovine serum albumin on ice for 30 min. Following removal of excess unbound antibody, aliquots of 10^6^ cells were shifted to 37°C for various periods of time. Then, each sample was split into two aliquots, which were diluted with DMEM under neutral (pH 7.4) or acid (pH 2) conditions and incubated for 1 min on ice. Samples were then washed and fluorescence measured with an LSRII flow cytometer (BD).

### Yeast-3-hybrid assays

SIVagmSab Nef proteins were fused to the GAL4 DNA-binding domain in the pBridge vector (Clontech) also expressing the σ2 subunit of AP-2 (a kind gift of Juan Bonifacino). The α subunit of rat AP-2 was cloned into pVP16/HA [58]. The protocol used subsequently has been previously described [36], [37] except that *Saccharomyces cerevisiae* strain Y190 was used and transformed using the Gietz lab kit (Molecular Research Reagents Inc.).

## Supporting Information

Figure S1 Mapping Tetherin antagonism determinants in Nef. (A and C) Quantitative fluorescence-based Western blot analysis of particle release. HIV-1 lacking Vpu and Nef was expressed together with the indicated Nef proteins (*in trans*) in the presence or absence of cpzTetherin. Virion lysates were probed with an anti-capsid antibody and p24 quantitation in virion samples was measured using LICOR. Average and standard deviation of 2-3 independent experiments is shown. (B and D) Cell lysates were probed with an anti-HIV-1 Nef antibody that also recognizes SIVcpzGb1 Nef. (E) Nef-mediated CD4 downregulation. TZMbl cells transfected (using Lipofectamine2000) with 1μg of pCG-IRES-GFP plasmids expressing various Nef proteins depicted in Figure 1. Cells were stained 48hrs post-transfection with a mouse anti-human CD4 antibody conjugated to Alexa700 (BD Pharmigen). Cell associated fluorescence in the 700nm and GFP channels were measured using an LSRII flow cytometer (BD). The median fluorescence intensity in the 700nm channel of GFP-positive cells is plotted. Average and standard deviation of 2-3 independent experiments is shown.
(TIF)

Figure S2 Identification of Nef amino acids important for Tetherin antagonism. (A) Quantitative fluorescence-based Western blot analysis of particle release. HIV-1 lacking Vpu and Nef was expressed together with the Nef indicated proteins (*in trans*) in the presence or absence of cpzTetherin. Virion lysates were probed with an anti-capsid antibody and p24 quantitation in virion samples was measured using LICOR. Average and standard deviation of 3 independent experiments is shown. (B) Cell lysates were probed with an anti-HIV-1 Nef antibody that also recognizes SIVcpzGb1 Nef. (C) TZMbl cells transfected (using Lipofectamine2000) with 1μg of pCG-IRES-GFP plasmids expressing various Nef proteins depicted in Figure 1. Cells were stained 48hrs post-transfection with a mouse anti-human CD4 antibody conjugated to Alexa700 (BD Pharmigen). Cell associated fluorescence in the 700nm and GFP channels were measured using an LSRII flow cytometer (BD). The median fluorescence intensity in the 700nm channel of GFP-positive cells is plotted. Average and standard deviation of 2-3 independent experiments is shown.
(TIF)

Figure S3 Mutation of Nef residues required for AP-2 binding abolishes Tetherin antagonism. (A) Quantitative Western blot analysis of particle release for SIV_MAC_ based viruses lacking Nef, co-expressed with the indicated wild type or mutant SIV_MAC_ Nef proteins fused to Venus (*in trans*) in the presence or absence of rhTetherin. Virion lysates were probed with an anti-HIV-1-capsid antibody and p24 in virion samples was quantitated using LICOR. Average and standard deviation of 2-3 independent experiments is shown. (B) Quantitative Western blot analysis of particle release for HIV-1 based viruses lacking Vpu and Nef, co-expressed with wild type or mutant SIVagmSab Nef proteins fused to Venus (*in trans*) in the presence or absence of agmTetherin. Virion lysates were probed with an anti-HIV-1-capsid antibody and p24 in virion samples was quantitated using LICOR. Average and standard deviation of 2-3 independent experiments is shown. (C) Quantitative Western blot analysis of particle release for HIV-1 based viruses lacking Vpu and expressing no Nef or wild type or mutant SIVcpzGb1 Nef (*in cis*) in the presence or absence of cpzTetherin. Virion lysates were probed with an anti-HIV-1-capsid antibody and p24 in virion samples was quantitated using LICOR. Average and standard deviation of 2-3 independent experiments is shown.
(TIF)

Figure S4 Nef-AP-2 co-localization in mammalian cells. Immunofluorescence analysis of cells transfected with the indicated Nef-Venus fusion (shown in green) protein (for details see Materials and Methods). Endogenous AP-2 was detected using an antibody against the AP-2 α subunit followed by an Alexa594 secondary (shown in red). Representative images of the cell in contact with the coverslip for each Nef are shown.
(TIF)

Figure S5 Inhibition of particle release in cell lines stably expressing moTetherin chimeras. (A) Schematic representation of the chimeric Tetherin proteins used in this study. The drawing outlines the structural organization of Tetherin in the lipid bilayer (black lines with gray filling) with the top being the extracellular medium and the bottom the intracellular milieu. Purple shapes represent moTetherin-derived regions, black lines represent huTetherin-derived regions and teal lines represent cpzTetherin-derived regions. (B) Quantitative Western blot analysis of virion release from cells stably expressing chimeric moTetherin containing the hu-Tetherin CT or the hu-Tetherin CT with a 5 amino acid (GDIWK) insertion. Cells were infected with VSV-G pseudotyped HIV-1 based viruses (pBRHIV-1NL4-3ΔVpu) lacking Vpu and encoding the indicated Nef proteins. Cell and virion lysates were probed with an anti-capsid antibody. Numbers below each lane represent the measurement of p24 CA associated with released virus particles (LICOR). (C) Quantitative Western blot analysis of virion release from cells stably expressing chimeric moTetherin containing the hu- or cpz-Tetherin CT. Experiments were performed as described in (B).
(TIF)

Figure S6 Tetherin cell surface downregulation by Nef. Representative experiments used to generate the graphs in Figure 4. (A) Surface staining of cells stably expressing chimeric moTetherin containing the huTetherin CT that was either unmodified or included a 5 amino acid (GDIWK) insertion. Cells were infected with VSV-G pseudotyped HIV-1-based viral vector stocks (pCCGW) expressing the indicated Vpu or Nef protein linked to an IRES-GFP cassette. Control viruses were generated using pCCGW that did not express Vpu or Nef. At 48h post-infection, cells were stained for Tetherin expression with anti-mouse Tetherin antibody conjugated to APC. Cell associated fluorescence in the APC and GFP channels was measured using an LSRII flow cytometer (BD). (B) Surface staining of cells stably expressing chimeric moTetherin containing the hu- or cpz-Tetherin CT. Cells were infected and stained as in (A).
(TIF)

Figure S7 Nef enhances the rate of Tetherin internalization. Cells stably expressing hu- or cpz-moTetherin chimeras were transduced with vectors expressing no Nef or SIVcpzGb1 Nef wild type or DXXXLL/AXXXLL mutant. Cells were then stained with anti-moTetherin antibody at 4°C and shifted to 37°C. At the indicated times thereafter cells were washed at acid pH, that should remove all surface-bound antibody. The data is plotted as the proportion of the fluorescent intensity observed at each time point relative to the neutral pH washed, T = 0 sample.
(TIF)

Figure S8 AP-2 is required for Nef to antagonize Tetherin. (A) Quantitative Western blot analysis of virion release from cells transfected with the indicated siRNA, an HIV-1 provirus lacking Vpu (and expressing no Nef or SIVcpzGb1 Nef) and cpzTetherin or a control plasmid. Virion lysates were probed with an anti-capsid antibody and p24 was quantitated in virion samples (LICOR). Average and standard deviation of 2 independent experiments is shown. (B) Same as (A) but particle release by SIV_MAC_ or SIV_MAC_ΔNef transfected cells in the presence or absence of rhTetherin is shown.
(TIF)

Figure S9 Cell surface downregulation of mutant cpzY6A/Y8A-Tetherin by SIVcpzGb1 Nef. Cells stably expressing moTetherin containing the cpzTetherin CT and TM domains with mutations at residues Y6A and Y8A were transduced with HIV-based viral vectors (pCCGW) expressing Nef-IRES-GFP. Cell surface Tetherin staining was performed using an anti-mouse Tetherin antibody conjugated to APC. Median fluorescence in the APC channel of GFP positive cells is plotted relative to the median fluorescence of cells infected with a control empty vector (that does not express Vpu or Nef) which was set as 100%. Data is plotted as the mean and standard deviation of 2 independent experiments. Results obtained with cells stably expressing moTetherin containing the hu- or cpz-Tetherin CT are plotted for comparison.
(TIF)
