# Supplementary figures and images for: SIV Nef Proteins Recruit the AP-2 Complex to Antagonize Tetherin and Facilitate Virion Release
Source: PLoS Pathog. 2011 May 19;7(5):e1002039. doi: 10.1371/journal.ppat.1002039 (PMC3098198; doi:10.1371/journal.ppat.1002039)

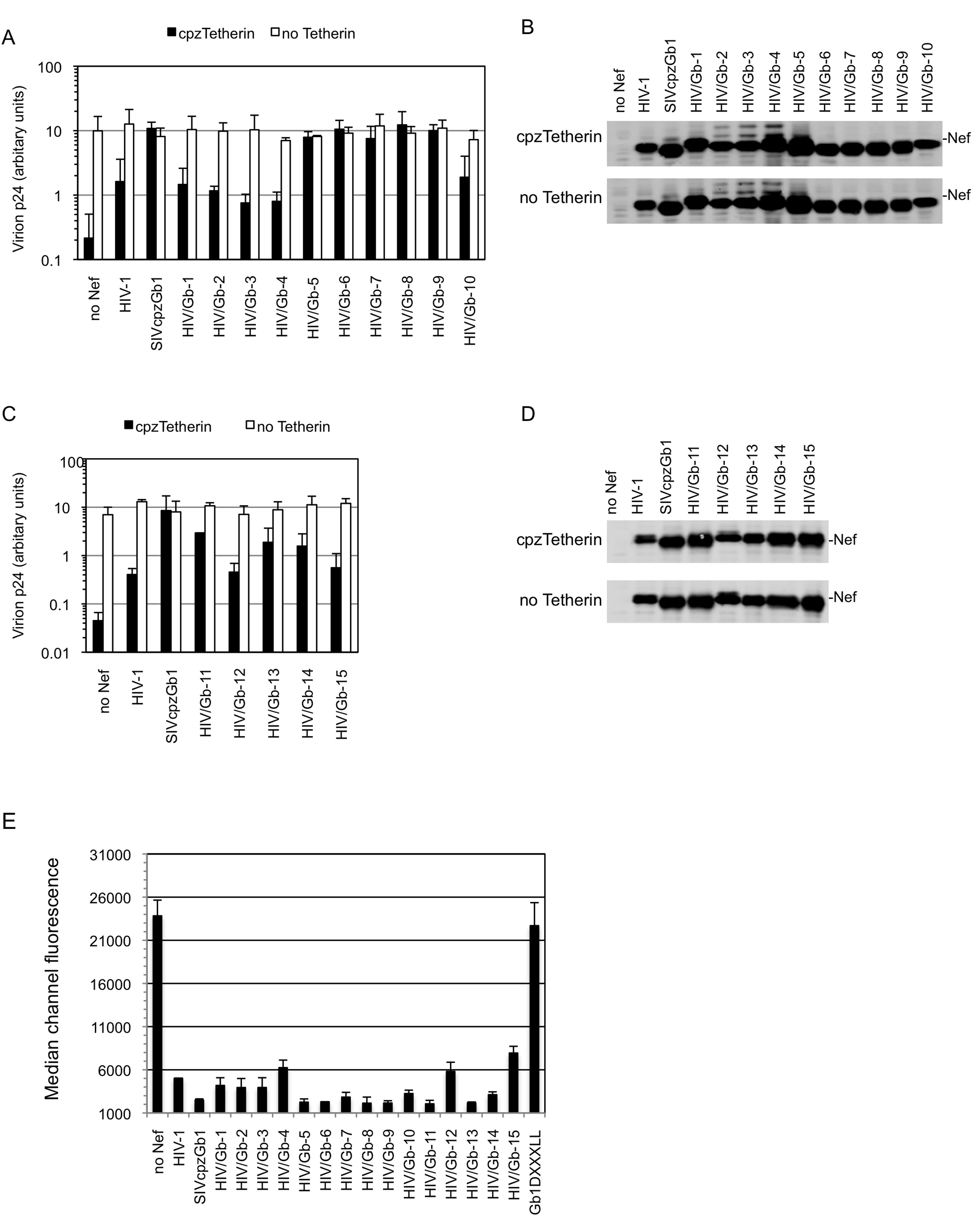

Supplement: Figure S1 — Mapping Tetherin antagonism determinants in Nef. (A and C) Quantitative fluorescence-based Western blot analysis of particle release. HIV-1 lacking Vpu and Nef was expressed together with the indicated Nef proteins (in trans) in the presence or absence of cpzTetherin. Virion lysates were probed with an anti-capsid antibody and p24 quantitation in virion samples was measured using LICOR. Average and standard deviation of 2-3 independent experiments is shown. (B and D) Cell lysates were probed with an anti-HIV-1 Nef antibody that also recognizes SIVcpzGb1 Nef. (E) Nef-mediated CD4 downregulation. TZMbl cells transfected (using Lipofectamine2000) with 1μg of pCG-IRES-GFP plasmids expressing various Nef proteins depicted in Figure 1. Cells were stained 48hrs post-transfection with a mouse anti-human CD4 antibody conjugated to Alexa700 (BD Pharmigen). Cell associated fluorescence in the 700nm and GFP channels were measured using an LSRII flow cytometer (BD). The median fluorescence intensity in the 700nm channel of GFP-positive cells is plotted. Average and standard deviation of 2-3 independent experiments is shown. (TIF) [file ppat.1002039.s001.tif]

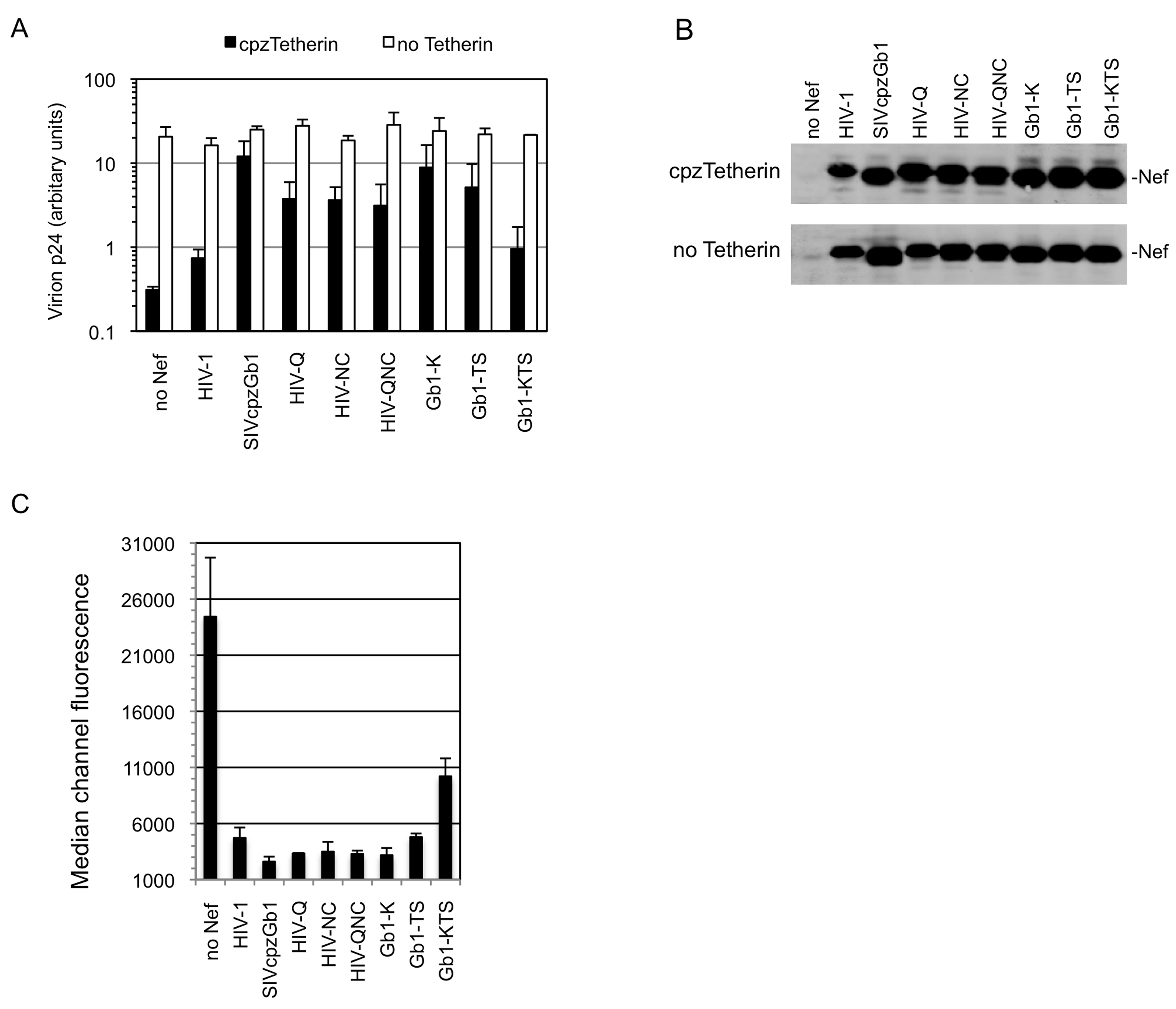

Supplement: Figure S2 — Identification of Nef amino acids important for Tetherin antagonism. (A) Quantitative fluorescence-based Western blot analysis of particle release. HIV-1 lacking Vpu and Nef was expressed together with the Nef indicated proteins (in trans) in the presence or absence of cpzTetherin. Virion lysates were probed with an anti-capsid antibody and p24 quantitation in virion samples was measured using LICOR. Average and standard deviation of 3 independent experiments is shown. (B) Cell lysates were probed with an anti-HIV-1 Nef antibody that also recognizes SIVcpzGb1 Nef. (C) TZMbl cells transfected (using Lipofectamine2000) with 1μg of pCG-IRES-GFP plasmids expressing various Nef proteins depicted in Figure 1. Cells were stained 48hrs post-transfection with a mouse anti-human CD4 antibody conjugated to Alexa700 (BD Pharmigen). Cell associated fluorescence in the 700nm and GFP channels were measured using an LSRII flow cytometer (BD). The median fluorescence intensity in the 700nm channel of GFP-positive cells is plotted. Average and standard deviation of 2-3 independent experiments is shown. (TIF) [file ppat.1002039.s002.tif]

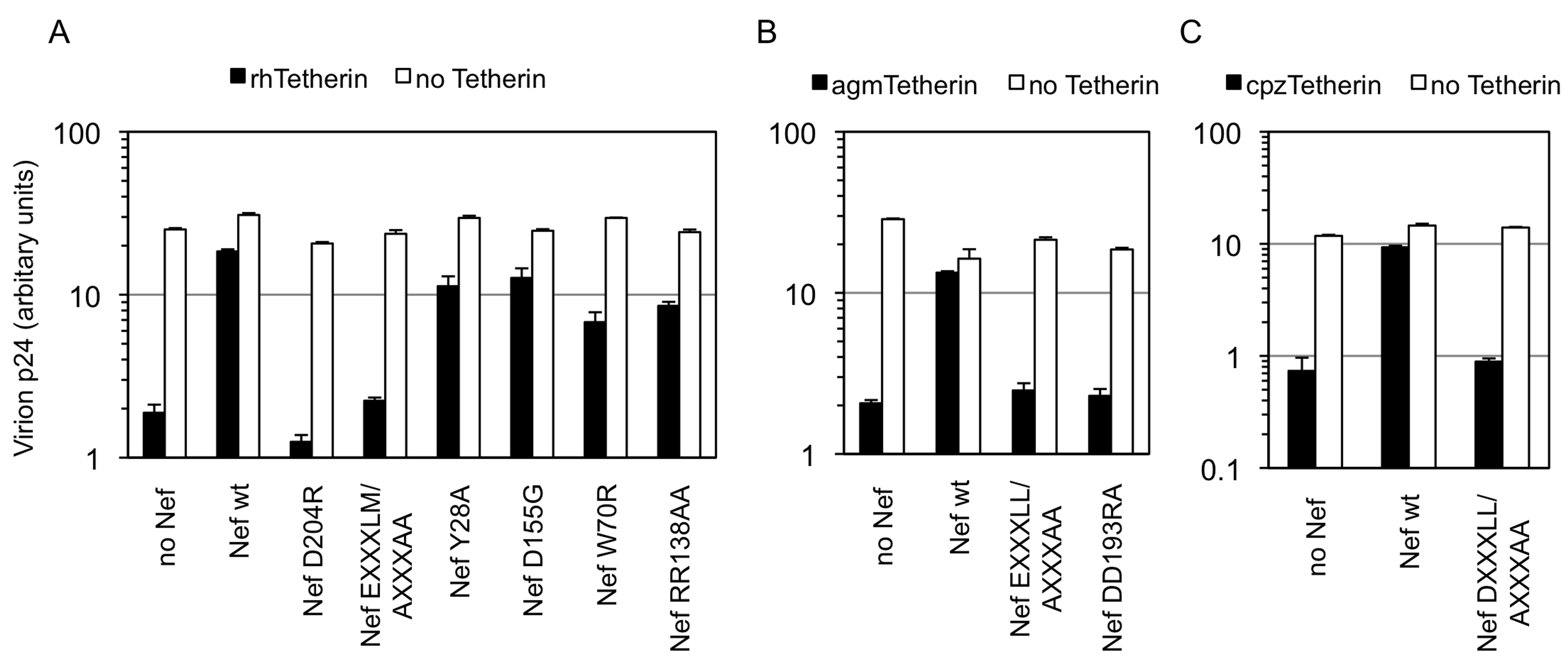

Supplement: Figure S3 — Mutation of Nef residues required for AP-2 binding abolishes Tetherin antagonism. (A) Quantitative Western blot analysis of particle release for SIVMAC based viruses lacking Nef, co-expressed with the indicated wild type or mutant SIVMAC Nef proteins fused to Venus (in trans) in the presence or absence of rhTetherin. Virion lysates were probed with an anti-HIV-1-capsid antibody and p24 in virion samples was quantitated using LICOR. Average and standard deviation of 2-3 independent experiments is shown. (B) Quantitative Western blot analysis of particle release for HIV-1 based viruses lacking Vpu and Nef, co-expressed with wild type or mutant SIVagmSab Nef proteins fused to Venus (in trans) in the presence or absence of agmTetherin. Virion lysates were probed with an anti-HIV-1-capsid antibody and p24 in virion samples was quantitated using LICOR. Average and standard deviation of 2-3 independent experiments is shown. (C) Quantitative Western blot analysis of particle release for HIV-1 based viruses lacking Vpu and expressing no Nef or wild type or mutant SIVcpzGb1 Nef (in cis) in the presence or absence of cpzTetherin. Virion lysates were probed with an anti-HIV-1-capsid antibody and p24 in virion samples was quantitated using LICOR. Average and standard deviation of 2-3 independent experiments is shown. (TIF) [file ppat.1002039.s003.tif]

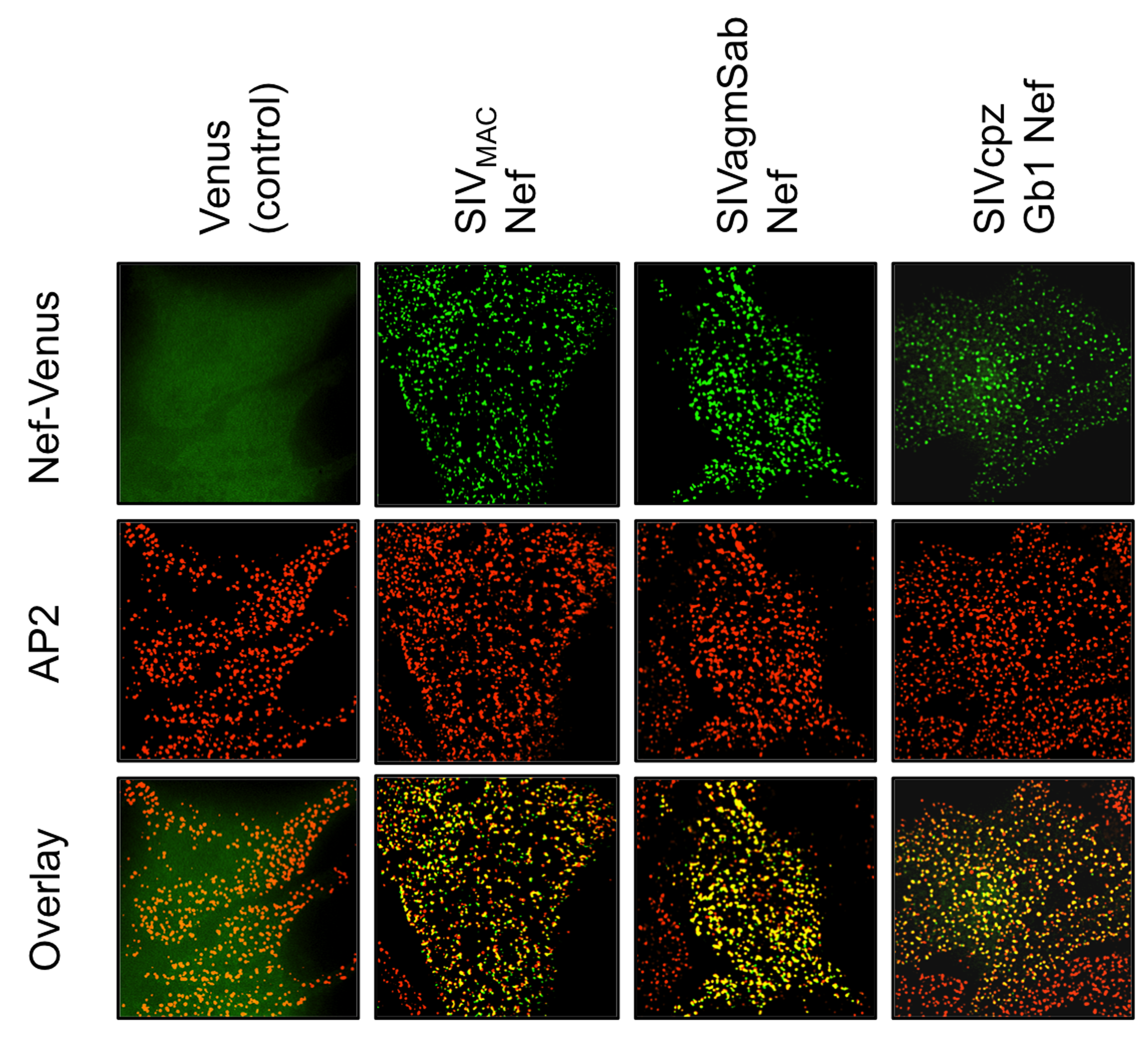

Supplement: Figure S4 — Nef-AP-2 co-localization in mammalian cells. Immunofluorescence analysis of cells transfected with the indicated Nef-Venus fusion (shown in green) protein (for details see Materials and Methods). Endogenous AP-2 was detected using an antibody against the AP-2 α subunit followed by an Alexa594 secondary (shown in red). Representative images of the cell in contact with the coverslip for each Nef are shown. (TIF) [file ppat.1002039.s004.tif]

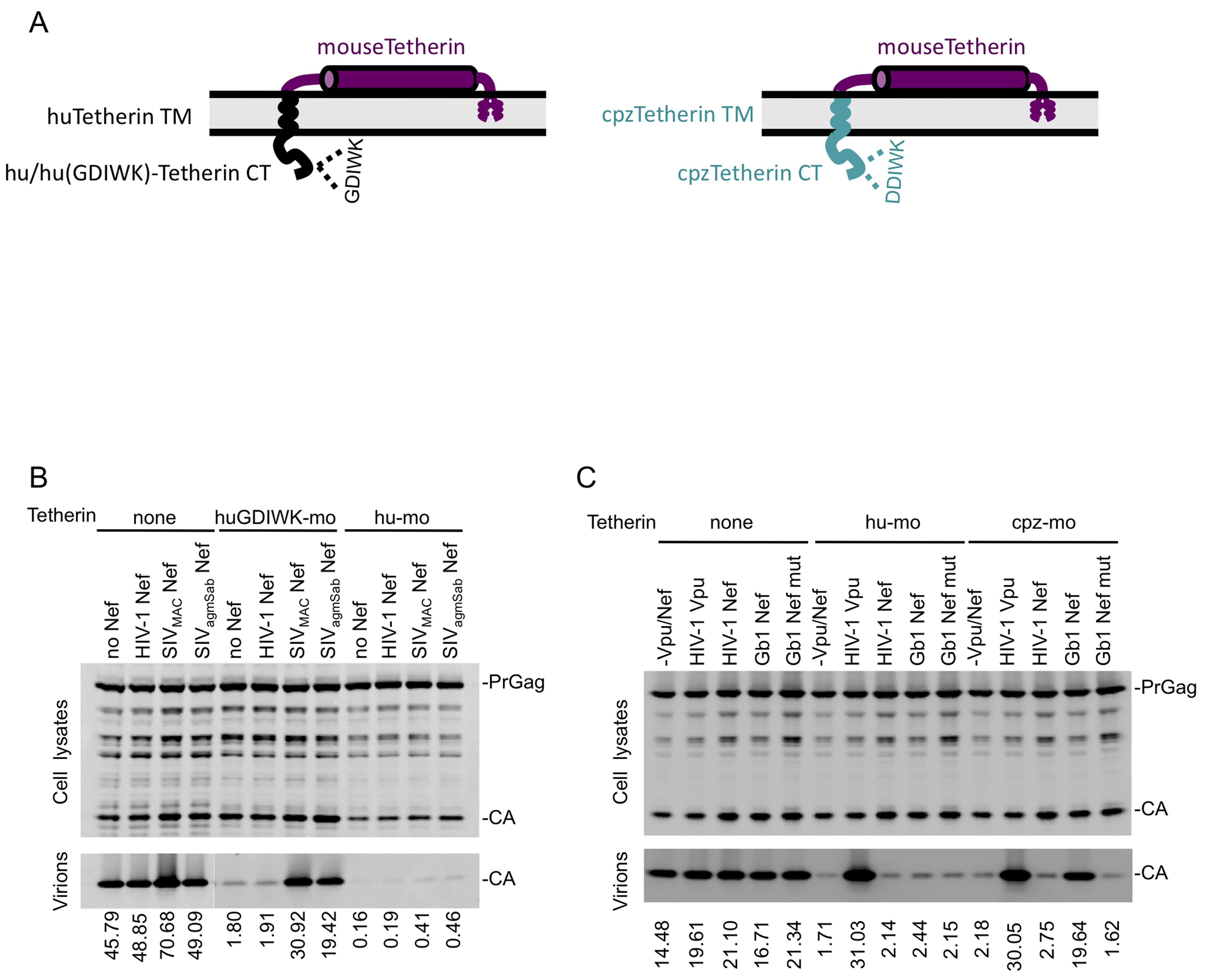

Supplement: Figure S5 — Inhibition of particle release in cell lines stably expressing moTetherin chimeras. (A) Schematic representation of the chimeric Tetherin proteins used in this study. The drawing outlines the structural organization of Tetherin in the lipid bilayer (black lines with gray filling) with the top being the extracellular medium and the bottom the intracellular milieu. Purple shapes represent moTetherin-derived regions, black lines represent huTetherin-derived regions and teal lines represent cpzTetherin-derived regions. (B) Quantitative Western blot analysis of virion release from cells stably expressing chimeric moTetherin containing the hu-Tetherin CT or the hu-Tetherin CT with a 5 amino acid (GDIWK) insertion. Cells were infected with VSV-G pseudotyped HIV-1 based viruses (pBRHIV-1NL4-3ΔVpu) lacking Vpu and encoding the indicated Nef proteins. Cell and virion lysates were probed with an anti-capsid antibody. Numbers below each lane represent the measurement of p24 CA associated with released virus particles (LICOR). (C) Quantitative Western blot analysis of virion release from cells stably expressing chimeric moTetherin containing the hu- or cpz-Tetherin CT. Experiments were performed as described in (B). (TIF) [file ppat.1002039.s005.tif]

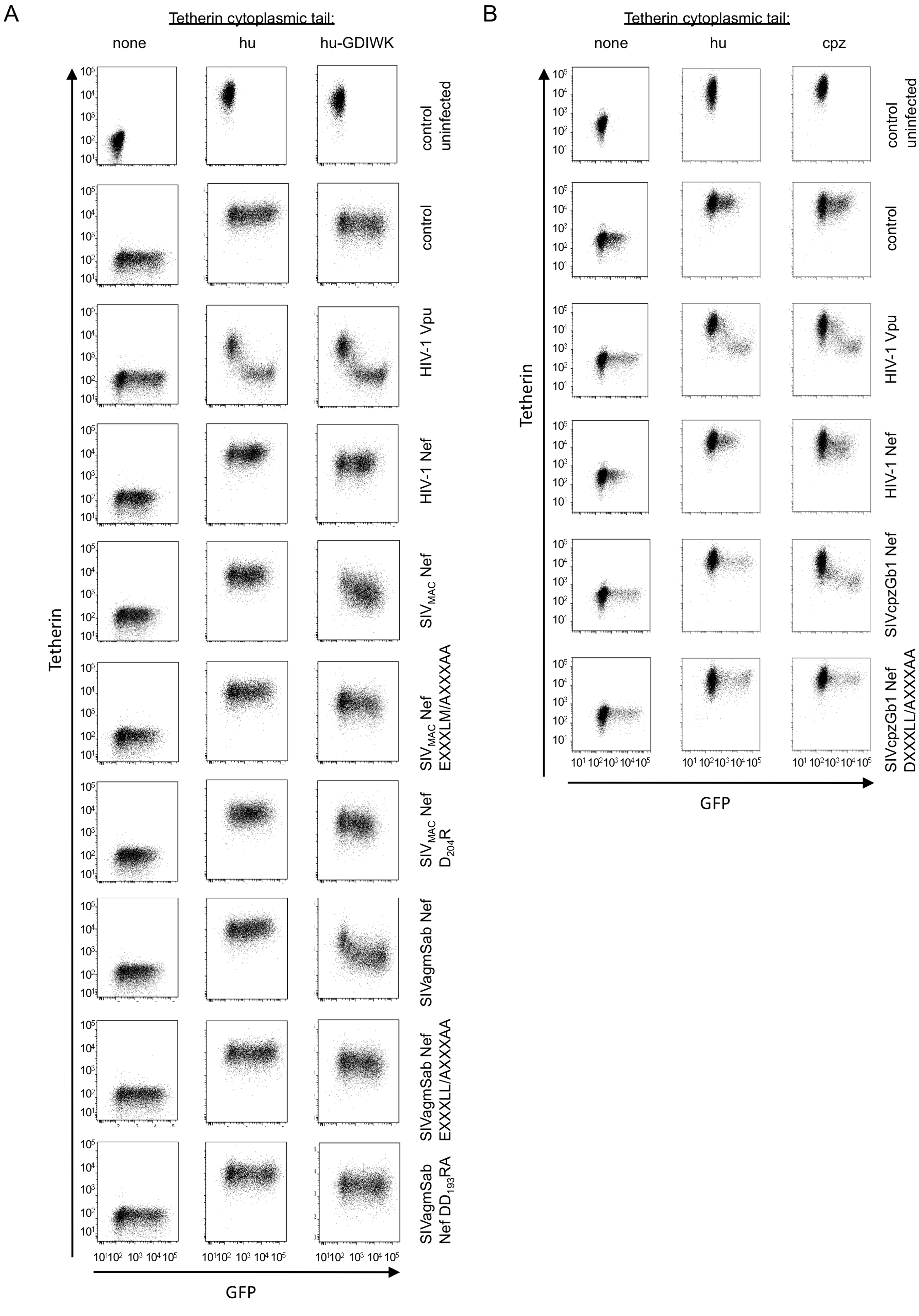

Supplement: Figure S6 — Tetherin cell surface downregulation by Nef. Representative experiments used to generate the graphs in Figure 4. (A) Surface staining of cells stably expressing chimeric moTetherin containing the huTetherin CT that was either unmodified or included a 5 amino acid (GDIWK) insertion. Cells were infected with VSV-G pseudotyped HIV-1-based viral vector stocks (pCCGW) expressing the indicated Vpu or Nef protein linked to an IRES-GFP cassette. Control viruses were generated using pCCGW that did not express Vpu or Nef. At 48h post-infection, cells were stained for Tetherin expression with anti-mouse Tetherin antibody conjugated to APC. Cell associated fluorescence in the APC and GFP channels was measured using an LSRII flow cytometer (BD). (B) Surface staining of cells stably expressing chimeric moTetherin containing the hu- or cpz-Tetherin CT. Cells were infected and stained as in (A). (TIF) [file ppat.1002039.s006.tif]

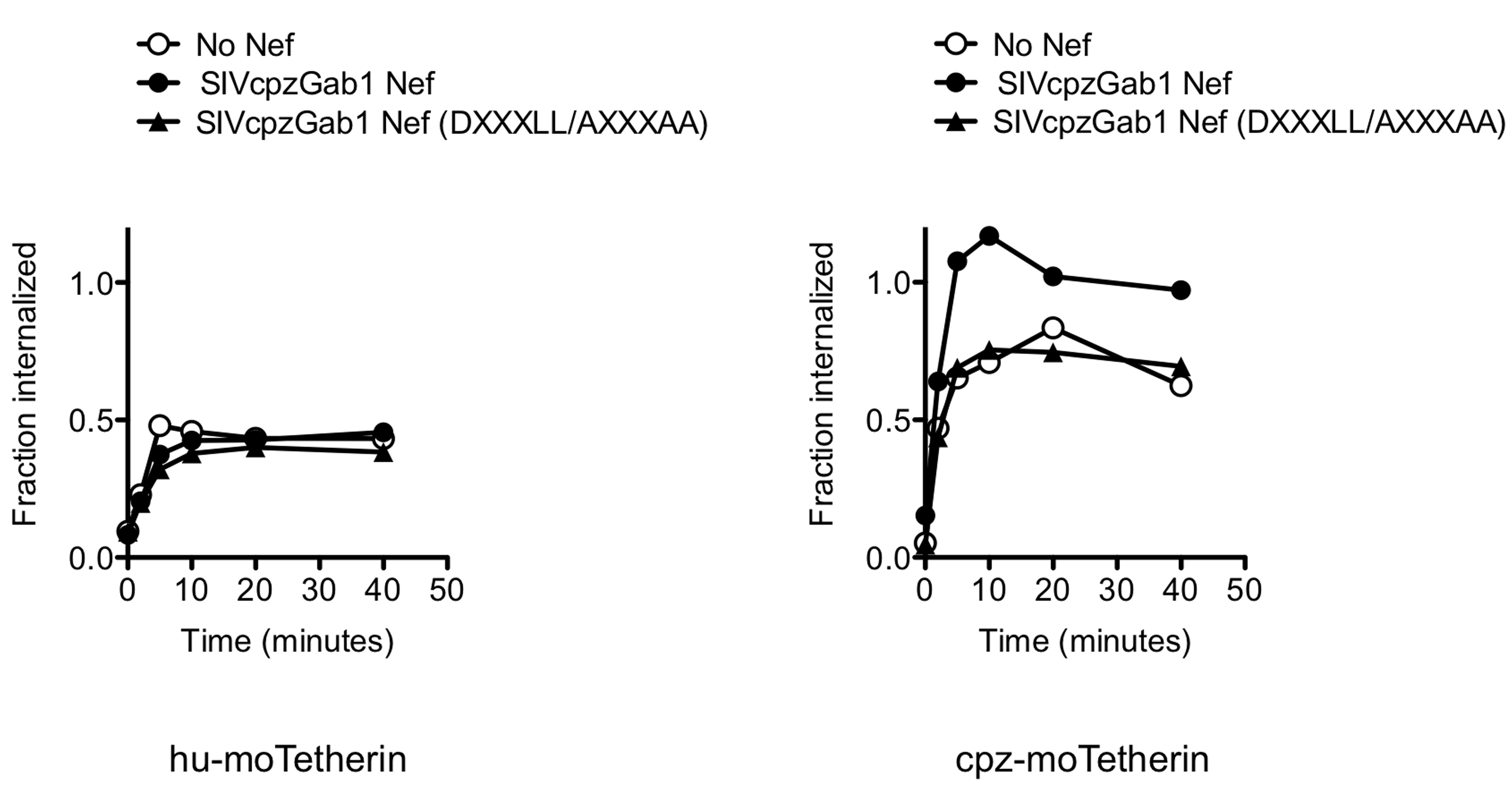

Supplement: Figure S7 — Nef enhances the rate of Tetherin internalization. Cells stably expressing hu- or cpz-moTetherin chimeras were transduced with vectors expressing no Nef or SIVcpzGb1 Nef wild type or DXXXLL/AXXXLL mutant. Cells were then stained with anti-moTetherin antibody at 4°C and shifted to 37°C. At the indicated times thereafter cells were washed at acid pH, that should remove all surface-bound antibody. The data is plotted as the proportion of the fluorescent intensity observed at each time point relative to the neutral pH washed, T = 0 sample. (TIF) [file ppat.1002039.s007.tif]

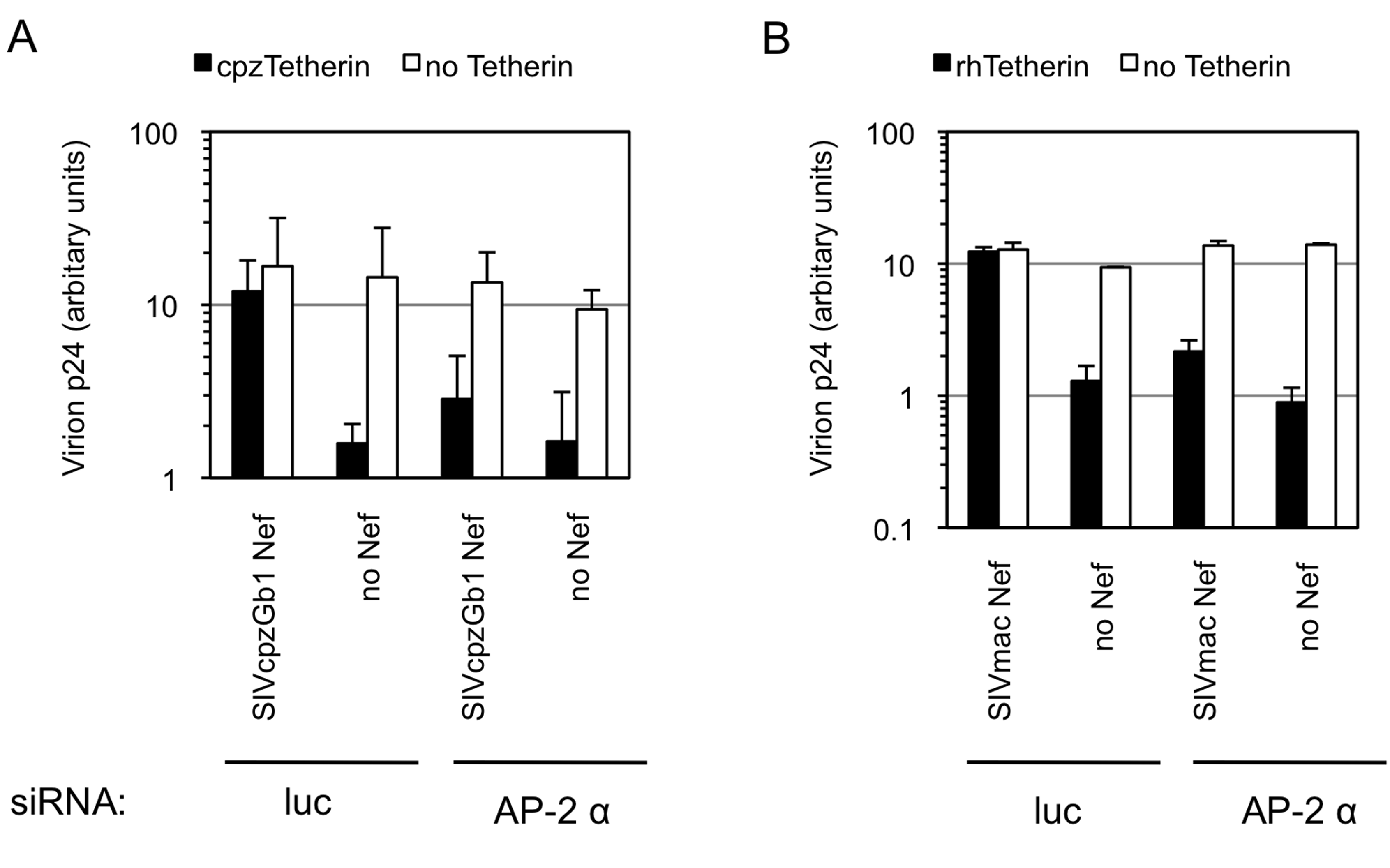

Supplement: Figure S8 — AP-2 is required for Nef to antagonize Tetherin. (A) Quantitative Western blot analysis of virion release from cells transfected with the indicated siRNA, an HIV-1 provirus lacking Vpu (and expressing no Nef or SIVcpzGb1 Nef) and cpzTetherin or a control plasmid. Virion lysates were probed with an anti-capsid antibody and p24 was quantitated in virion samples (LICOR). Average and standard deviation of 2 independent experiments is shown. (B) Same as (A) but particle release by SIVMAC or SIVMACΔNef transfected cells in the presence or absence of rhTetherin is shown. (TIF) [file ppat.1002039.s008.tif]

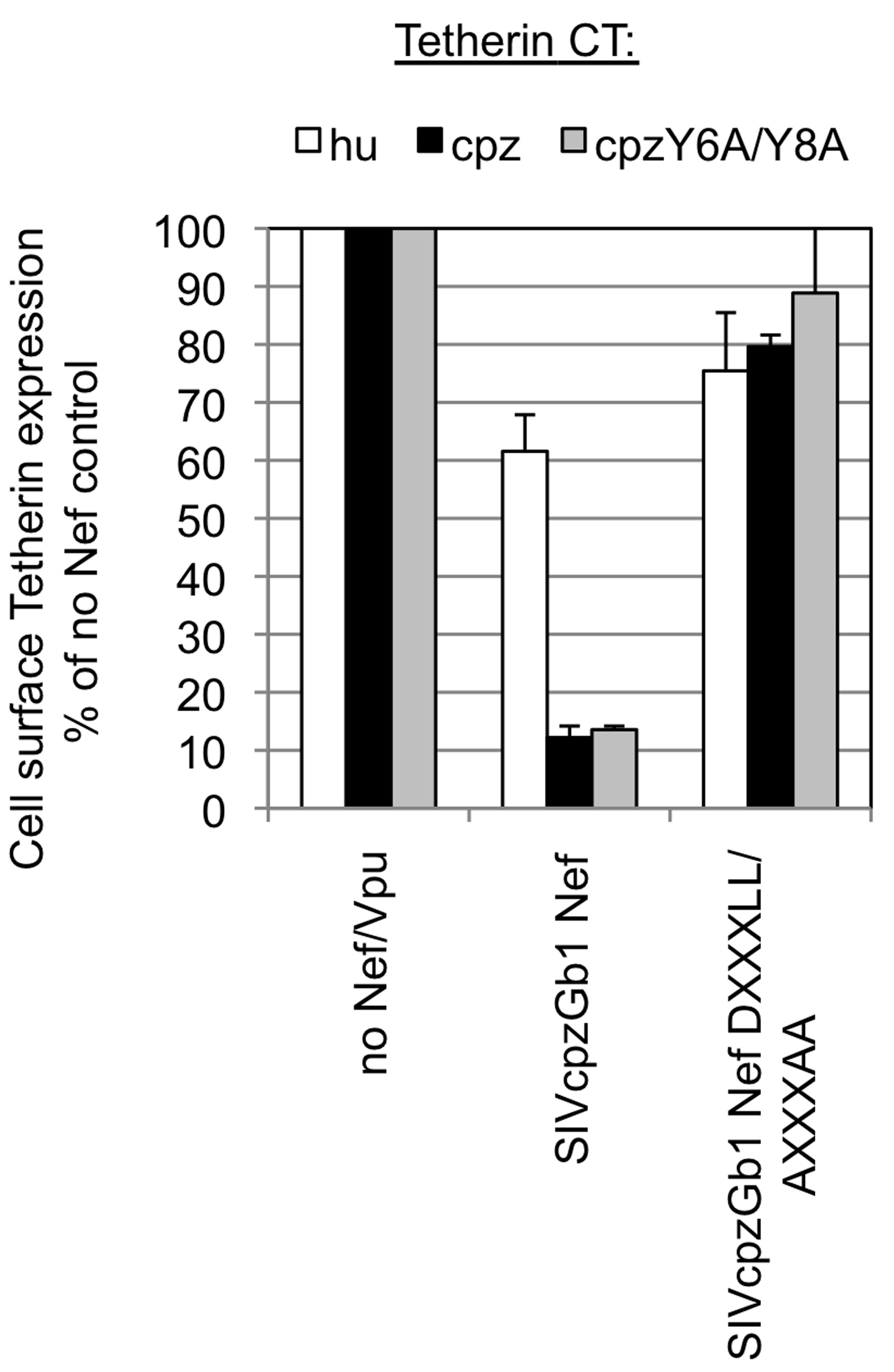

Supplement: Figure S9 — Cell surface downregulation of mutant cpzY6A/Y8A-Tetherin by SIVcpzGb1 Nef. Cells stably expressing moTetherin containing the cpzTetherin CT and TM domains with mutations at residues Y6A and Y8A were transduced with HIV-based viral vectors (pCCGW) expressing Nef-IRES-GFP. Cell surface Tetherin staining was performed using an anti-mouse Tetherin antibody conjugated to APC. Median fluorescence in the APC channel of GFP positive cells is plotted relative to the median fluorescence of cells infected with a control empty vector (that does not express Vpu or Nef) which was set as 100%. Data is plotted as the mean and standard deviation of 2 independent experiments. Results obtained with cells stably expressing moTetherin containing the hu- or cpz-Tetherin CT are plotted for comparison. (TIF) [file ppat.1002039.s009.tif]
